# Wildmeat consumption and child health in Amazonia

**DOI:** 10.1038/s41598-022-09260-3

**Published:** 2022-04-06

**Authors:** Patricia Carignano Torres, Carla Morsello, Jesem D. Y. Orellana, Oriana Almeida, André de Moraes, Erick A. Chacón-Montalván, Moisés A. T. Pinto, Maria G. S. Fink, Maíra P. Freire, Luke Parry

**Affiliations:** 1 Programa de Pós-Graduação em Modelagem de Sistemas Complexos, Escola de Artes, Ciências e Humanidades, EACH, Universidade de São Paulo, São Paulo, Brazil; 2 Instituto Leônidas e Maria Deane, Fundação Oswaldo Cruz, Manaus, Brazil; 3 Núcleo de Altos Estudos Amazônicos, Universidade Federal do Pará, Belém, Brazil; 4 Manaus, Brazil; 5 Department of Mathematics and Statistics, Lancaster University, Lancaster, UK; 6 Lancaster Environment Centre, Lancaster University, Lancaster, UK

**Keywords:** Conservation biology, Ecosystem services, Sustainability

## Abstract

Consuming wildmeat may protect against iron-deficiency anemia, a serious public health problem globally. Contributing to debates on the linkages between wildmeat and the health of forest-proximate people, we investigate whether wildmeat consumption is associated with hemoglobin concentration in rural and urban children (< 5 years old) in central Brazilian Amazonia. Because dietary practices mediate the potential nutritional benefits of wildmeat, we also examined whether its introduction into children’s diets is influenced by rural/urban location or household socio-economic characteristics. Sampling 610 children, we found that wildmeat consumption is associated with higher hemoglobin concentration among the rural children most vulnerable to poverty, but not in the least vulnerable rural, or urban children. Rural caregivers share wildmeat with children earlier-in-life than urban caregivers, potentially because of cultural differences, lower access to domesticated meat, and higher wildmeat consumption by rural households (four times the urban average). If wildmeat becomes unavailable through stricter regulations or over-harvesting, we predict a ~ 10% increased prevalence of anemia among extremely poor rural children. This modest protective effect indicates that ensuring wildmeat access is, alone, insufficient to control anemia. Sustainable wildlife management could enhance the nutritional benefits of wildlife for vulnerable Amazonians, but reducing multidimensional poverty and improving access to quality healthcare are paramount.

## Introduction

Researchers and policy-makers have long been interested in the role of wildmeat in supporting the food and nutrition security of people living in and around tropical forests^1^. However, empirical research into wildmeat’s contribution to human health and nutrition remains limited^2,3^. This research gap is problematic because reliable evidence is vital to inform debates on the ethics and health implications of policies that promote the sustainable use of wildlife by forest-proximate people, including in the tropics. Many wildlife species are threatened by legal or illegal hunting^3,4^ and it has been long argued that wildmeat harvest is causing widespread defaunation of tropical forests, the so-called ‘bushmeat crisis’^1^. Evaluating wildmeat’s contribution to health and nutrition is also urgent given contentious proposals that the human impacts of zoonotic diseases, such as COVID-19 and SARS, justify more draconian governance of wildmeat harvest and consumption^5^. Conversely, many scientists oppose any ban on wildmeat consumption and instead advocating a more measured response to COVID-19^5,6^, in part because wildmeat provides critical resources for the world’s most vulnerable people. Summarizing, decision-makers require evidence in order to balance the needs, rights, and health of forest-dwellers against the ecological risks of unsustainable hunting, and health risks of zoonotic disease^7,8^.

Proponents of sustainable hunting emphasize prevalent wildmeat consumption among indigenous peoples and other rural populations, related to the cultural significance of hunting and the necessity driven by poverty, and limited access to alternative meat sources^8^. Numerous studies show that wildmeat makes an important contribution to protein intake, especially in Africa and locations with limited access to other meat sources^10–13^. In Nigerian forests, wildmeat consumption has been associated with lower food insecurity in poor rural communities^9^. Another study demonstrated that wildmeat contributes to dietary fat intake among an indigenous community in the Ecuadorian Amazon^14^.

Recent research has moved beyond investigating rural populations to examine wildmeat consumption among urban dwellers. Indeed, in certain Congolese urban areas, wildmeat constitutes the majority of meat intake^13^, and its consumption can increase ingestion of protein, fat, and micronutrients^15,16^. Despite evidence that wildmeat provides important macro and micronutrients to rural and urban diets, there is only limited research into how wildmeat may support the nutritional status of forest-proximate people^3^. For instance, wildmeat consumption could be related to potential variation in linear growth during childhood, which is assessed using non-invasive anthropometric measures. Furthermore, research could test for a relationship between an adult or child’s wildmeat consumption and deficiencies in vitamin intake, or iron stores. For example, iron-deficiency anemia is diagnosed through clinical assessment, and by measuring blood hemoglobin concentration.

Wildmeat is argued to protect rural children in central Africa against chronic malnutrition, but this relationship has not been empirically tested^17^. Only one study, in a Malagasy village, has directly examined child health and wildmeat consumption, finding that consumption is positively correlated with hemoglobin concentration among children under 12 years-old^4^. However, it is still unclear whether consuming wildmeat reduces anemia risks in rural communities—and urban contexts—elsewhere in the forested tropics. Additionally, to understand the potential nutritional benefits of wildmeat for children, we need greater insights into household food practices. Young children are particularly vulnerable to malnutrition due to poor feeding practices^18^, including a failure to introduce sufficiently nutritious foods. Caregivers’ food practices are shaped by culture, socioeconomic differences, and habits acquired early-in-life^19,20^. Consequently, the age at which children begin to eat wildmeat may vary within a population and between rural and urban caregivers. This variation is important because the potential benefits for child health may be under-utilized if caregivers choose not to share wildmeat when eaten in the household.

In this paper, we assess the role of wildmeat in supporting child health among rural and urban Amazonians. We use the term ‘wildmeat’ to refer to terrestrial game species, excluding the meat from aquatic animals (e.g., fishes, turtles). We aim to evaluate whether wildmeat consumption potentially protects young children against anemia, to determine which kinds of forest-proximate children (rural or urban, and in each location, the sub-populations most or least vulnerable to poverty) may benefit, and to assess whether sharing wildmeat with children is influenced by location (rural/urban), household characteristics, and in urban areas, caregivers’ origin (specifically, whether or not they are rural–urban migrants).

Whether from domesticated or wild animals, animal source foods (ASFs) are likely to support the health of forest-proximate children in numerous ways. For instance, ASFs are important for children’s physical and cognitive development^21,22^. Our study focuses on the potential benefits of wildmeat in reducing the burden of anemia among Amazonian children. Insufficient iron intake can lead to iron-deficiency anemia (IDA) (herein, anemia), which is more likely for children whose diets are low in ASFs^23^. IDA is not the only form of anemia, but iron deficiency is the major cause of anemia in children^24^. IDA is also the leading global cause of disability in children under 5 years^25^, and the most prevalent micronutrient deficiency^24,26^. Anemia, regardless of severity, poses an enormous public health problem because it impairs childhood development in myriad ways, including depressing energy levels, growth, cognitive and motor skills, and socioemotional and neurophysiological functioning^27^. Childhood anemia is also linked to higher risks of illness, reduced educational performance, and lower productivity in adulthood, thus, perpetuating the cycle of poverty^26–28^.

The contribution of wildmeat to child health will depend on caregivers’ choices about when to begin sharing ASFs. During the weaning process, children in the Global South are typically fed only limited quantities of ASFs^29^, and caregivers may avoid sharing them with younger infants because they lack teeth for chewing^30^. Importantly, ASF feeding practices are socially determined, being shaped by maternal education, economic circumstances, and sociocultural norms and beliefs^30,31^. Whether a child is fed wildmeat, or not, will partly depend on a household’s access to different ASFs, related to socially-mediated access to wildmeat, proximity to urban markets, and the relative affordability of particular ASFs^4^. Consequently, the contribution of wildmeat to iron intake likely varies between and within rural and urban populations. In Amazonia, there is tentative evidence that, even in urban areas, eating more wildmeat is associated with greater intake of iron and other micronutrients^15^. Amazonian research also shows urban consumption of some wildlife species correlates with lower monetary income and rural–urban migrant households^32,33^, hinting at the nutritional importance of wildmeat for these vulnerable urban populations. Fish is consumed daily in many Amazonian households and provides adequate dietary protein but insufficient iron^35^. Beef contains more iron than chicken (after fish, chicken is the most frequently consumed ASF in Amazonia) but is unaffordable to typical urban or rural households in central Amazonia –mostly far from large-scale deforestation frontiers– and therefore rarely consumed^15,34^^.^

In Amazonia, deep social inequalities and dietary limitations contribute to poor health outcomes, particularly for marginalized populations. Rural diets in the region are based on starchy staples (mainly manioc) and fish, with limited consumption of fruits and vegetables^35,36^. In Amazonian towns, typical diets lack diversity, including only small quantities of fruits, vegetables, and meats. Such a diet translates into low intake of essential micronutrients (e.g., vitamins A and C, zinc, and iron), and impaired nutritional status among Amazonian children (e.g., stunting [short height-for-age])^37^. Together with the impoverished North-East, the Amazon region has Brazil’s highest prevalence of childhood anemia (> 30%)^37–40^ and the highest rate of child mortality from malnutrition (0.52 deaths per 10,000 children < 5 years old)^41^. Relative to other Brazilians, marginalized rural Amazonians (e.g., indigenous groups, non-tribal river-dwelling *ribeirinhos*, Afro-descendent *quilombolas*) experience stark health inequities, including the greatest risks of anemia. Childhood anemia prevalence among these groups exceeds 50% in some locations^42,43^, comparable with the highest rates in the world in sub-Saharan Africa, and far above the overall prevalence in South America (18.8%)^44^.

In this study, we investigate the relationship between wildmeat consumption and hemoglobin concentration (< 11 g/dL indicates anemia) in Amazonian children aged between 6 months and 5-years-old. Our novel contribution is examining this linkage in rural and urban populations, and assessing the determinants of when wildmeat is introduced to children’s diets. Specifically, household socioeconomic factors, and cultural origin (if urban caregivers migrated from rural areas), and rural versus urban location. We addressed these issues by sampling 610 children in four municipalities in central Brazilian Amazonia, capturing spatial and socioeconomic heterogeneity. The study municipalities are expansive (each 9,500–69,500 km^2^), highly river-dependent, and characterized by high forest cover (89.7–96.9% remaining), remoteness from large cities, and widespread multi-dimensional poverty, including food insecurity. The urban areas comprised three small towns (~ 7,000–15,000 inhabitants) and one medium-sized town (~ 30,000 inhabitants). Sampled riverine rural communities varied in their distance to towns. Our specific research questions were: (i) is the age at which children begin eating wildmeat affected by rural/urban location, household characteristics (caregiver’s education, household monetary income), and in urban areas, caregivers’ origin (i.e., if they self-identify as a rural–urban migrant)? Any effect of being a migrant is therefore marginal to the effects of (potentially) lower income and education, and, instead, is considered indicative of (unmeasured) sociocultural differences compared with other town-dwellers. Any variation in wildmeat-sharing practices of rural versus urban caregivers may reflect structural socioeconomic differences in access to different ASFs (either through market exchange or gifting, reciprocity, etc.), and potential cultural differences (i.e., wildmeat-sharing with children may be shaped by ways of life, which reflect particular values and traditions). (ii) Is wildmeat consumption associated with hemoglobin concentrations in rural and urban children, and does this vary between within-area subpopulations of households that are most or least vulnerable to poverty? We classified most and least vulnerable households based on monetary income (indicative of income poverty), and using a multidimensional poverty index (Poverty Probability Index-PPI^45^). Finally, we evaluated the potential effects of changing scenarios of wildmeat consumption on anemia prevalence among children in central Amazonia.

## Results

**Figure 1:**
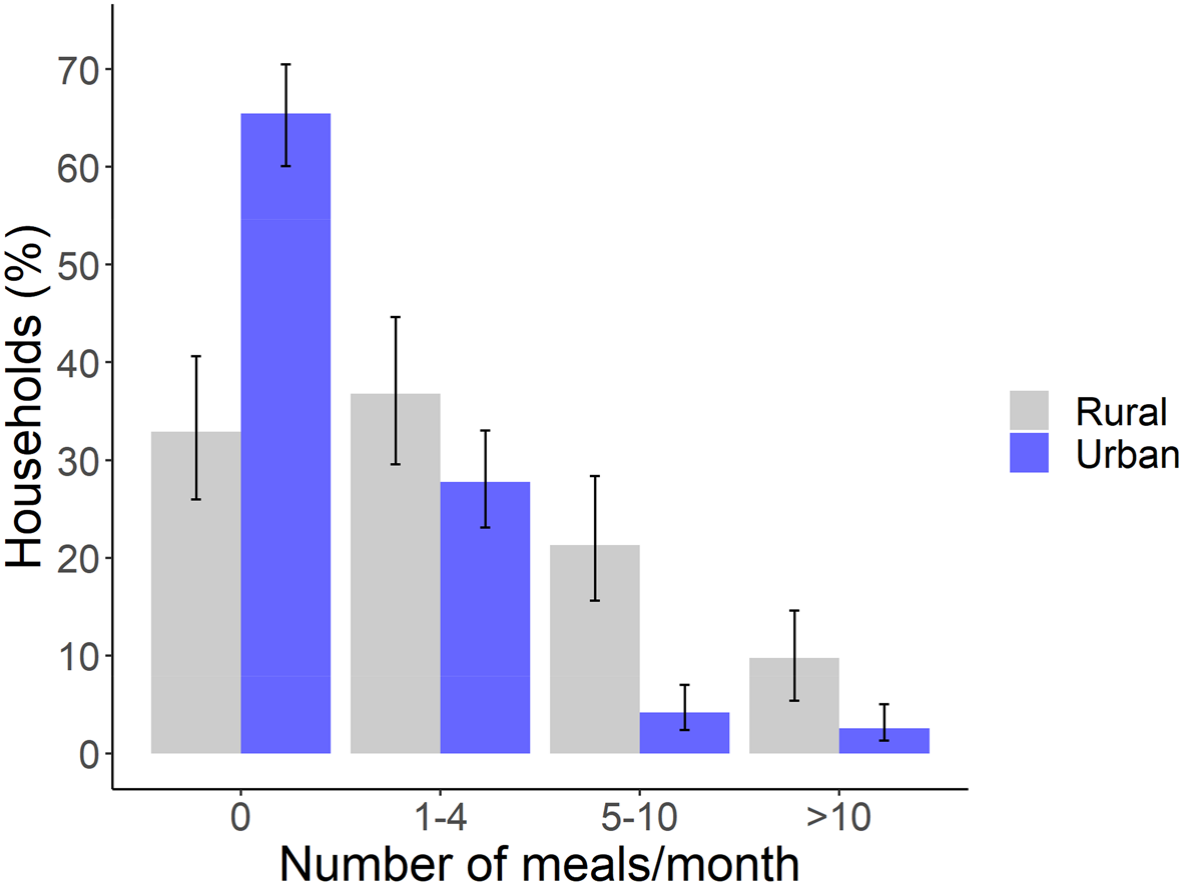
Frequency of wildmeat consumption by rural and urban Amazonian households, based on meals consumed within the previous 30 days. Only households where wildmeat was consumed in the previous 12 months are included. Error bars represent 95% CI, calculated using the Wilson score interval (using package ‘binom’ in R).

Rural households consumed wildmeat more frequently than urban households. Over two-thirds of rural households ate wildmeat at least monthly (69.5%), compared to 35.4% of urban households. Indeed, 36.4% of rural households ate wildmeat at least weekly (Fig. 1; Supplementary Fig. S1).

In rural households, wildmeat consumption (mean 1.7 days/week) was second only to fish (6.2 days/week; Table 1). Least vulnerable rural households consumed wildmeat and chicken more often than the most vulnerable households. Beef was seldom eaten in rural areas (overall, 0.2 days/week). In urban households, fish (3.2 days/week) was also the main ASF, followed by chicken (2.1 days/week), whereas wildmeat was consumed less often (0.3 days/week; Supplementary Fig. S2).

**Table 1:** Hemoglobin concentration, anemia prevalence and consumption of different types of animal source foods (ASF), among urban and rural children in Central Amazon.

Location	Vulnerability to poverty (subpopulations)	Mean Hb concentration (g/dL)^b^	Anemia prevalence (%)^c^	Household consumption of selected ASFs (mean days/week)			
				Wildmeat^d^	Fish^d^	Chicken^d^	Beef^d^
Urban	Most vulnerable^a^	11.00 (10.82–11.17)	47.1	0.32 (0.12–0.52)	3.10 (2.62–3.58)	1.87 (1.54–2.23)	0.86 (0.56–1.15)
	Least vulnerable^a^	11.14 (10.96–11.32)	42.1	0.27 (0.04–0.50)	3.38 (2.80–3.95)	2.44 (1.95–2.94)	1.31 (0.93–1.69)
	Whole urban sample	11.07 (10.95–11.20)	44.6	0.30 (0.15–0.44)	3.22 (2.86–3.59)	2.05 (1.75–2.34)	1.05 (0.82–1.29)
Rural	Most vulnerable^a^	10.51 (10.27–10.76)	60.6	1.05 (0.55–1.56)	6.39 (5.97–6.82)	0.61 (0.22–0.99)	0.09 (0.00–0.19)
	Least vulnerable^a^	10.72 (10.50–10.94)	56.9	2.40 (1.61–3.18)	6.04 (5.44–6.65)	1.07 (0.50–1.63)	0.22 (0.01–0.43)
	Whole rural sample	10.61 (10.45–10.78)	58.7	1.65 (1.19–2.11)	6.24 (5.88–6.59)	0.81 (0.48–1.14)	0.15 (0.05–0.26)

^a^Most and least vulnerable children were classified based on household monetary income being above or below the median of that location type (e.g., rural).

^b^Mean hemoglobin concentration (g/dL). 95% Confidence Intervals in parentheses.

^c^Anemic children were defined as having Hb < 11 g/dL.

^d^Mean number of days in which each type of meat was consumed in the previous 7 days. 95% Confidence Intervals in parentheses.

### Wildmeat in children’s diets

**Figure 2:**
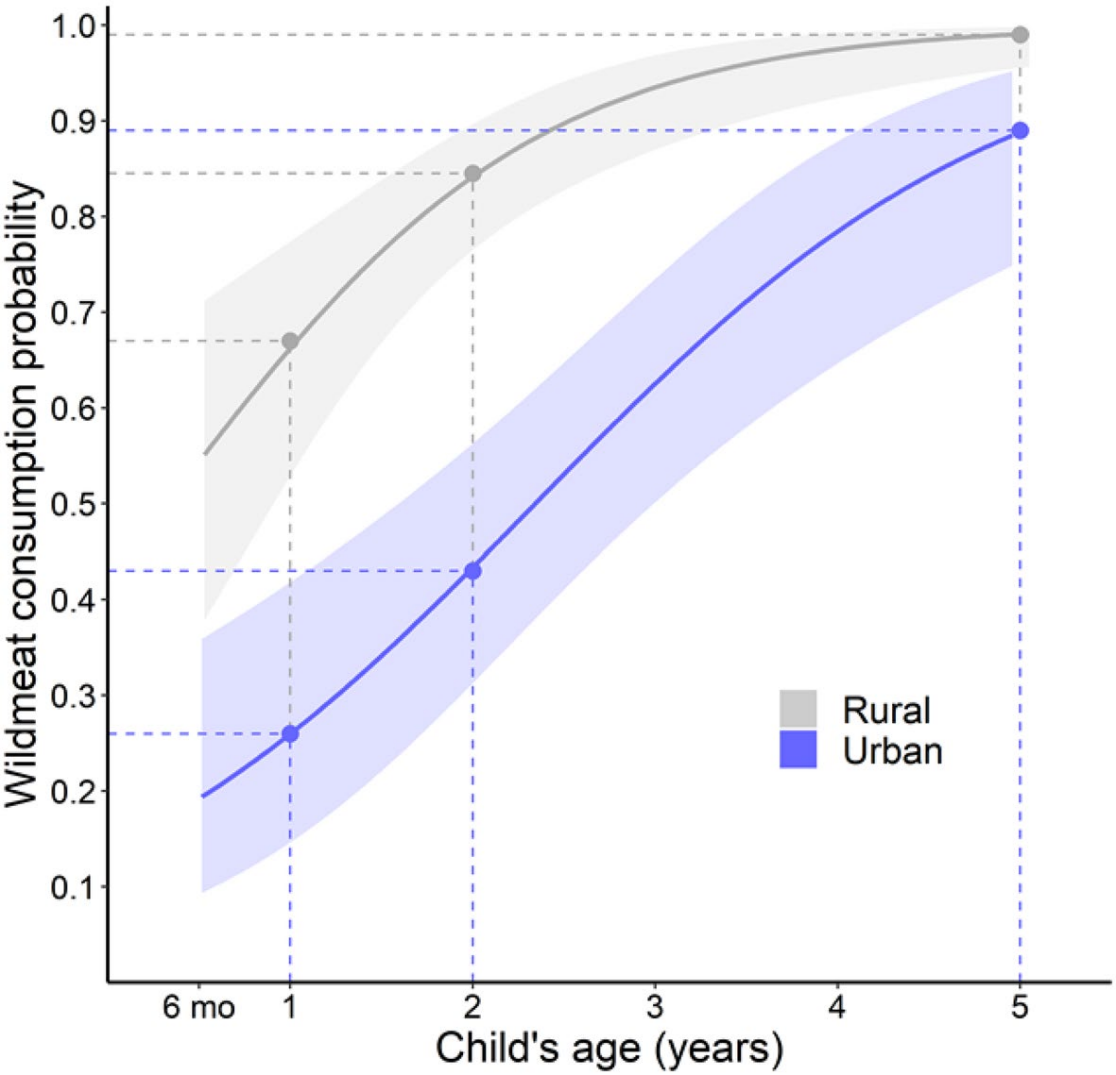
Increasing probability of wildmeat consumption with the age of Amazonian children in rural (gray) and urban (blue) areas, in households that consume wildmeat (those which consumed wildmeat in the previous 12 months). Urban children are those whose caregivers are not rural–urban migrants (Supplementary Fig. S1). The shaded areas represent 95% CI.

**Figure 3:**
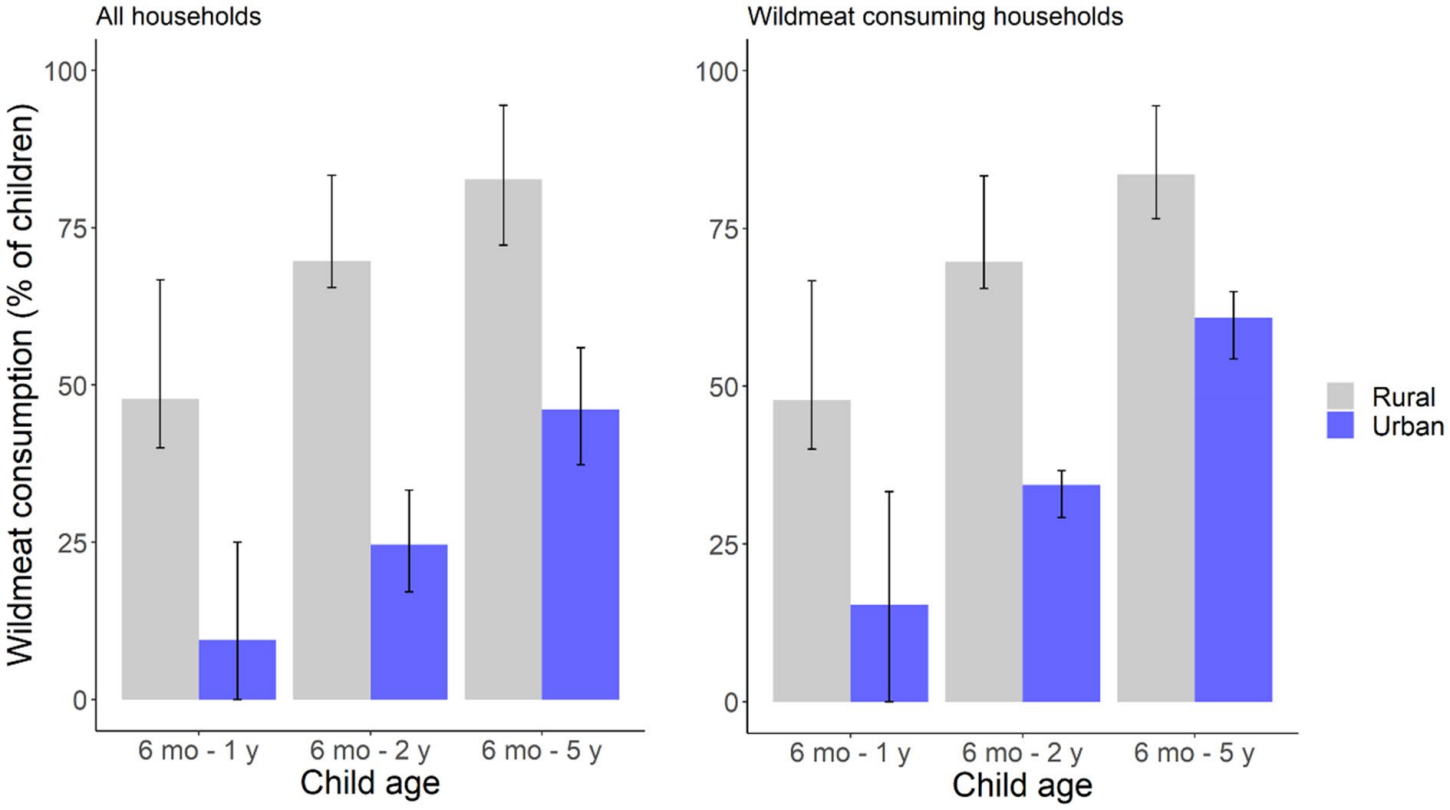
Percentage of Amazonian children that consume wildmeat in different stages of infancy and early childhood, separated by rural and urban locations. Wildmeat-consuming households are defined as those which consumed wildmeat in the previous 12 months. Error bars represent 95% CI, calculated using the Wilson score interval (using the R package ‘binom’).

In both rural and urban areas, the likelihood of consuming wildmeat (at least sometimes) increased with age; being one year older more than doubles the odds (range 2.19–2.75; CI: 1.26–4.26) (Supplementary Table S1, Supplementary Table S2). Rural children were much more likely to eat wildmeat than same-aged urban children, for two reasons. First, rural households typically ate wildmeat much more often. Second, rural caregivers were much more likely to share wildmeat with children of a certain age, compared to urban caregivers. In urban areas, children of a rural–urban migrant parent were more likely to eat wildmeat than children in non-migrant households, with mean odds 148% higher (CI = 1.26–4.85). In wildmeat-consuming rural households, the probability of sharing with a child was 0.67 (CI = 0.52–0.79) by one-year-old. This probability is comparable to a three-year-old in a non-migrant urban household (0.63; CI = 0.50–0.73) (Fig. 2) or a two-year-old child in a rural–urban migrant household (0.65; CI = 0.53–0.76) (Supplementary Fig. S3, Supplementary Table S2). Overall, only 17.1% of urban infants (< 1-year-old) ate wildmeat (CI = 8.5–31.3), compared to 50% (CI = 32.1–67.9) of rural infants. Urban children remained much less likely to consume wildmeat throughout early childhood. Hence, 62% of urban children aged two-to-five years old (CI = 55.9–67.8) sometimes ate wildmeat, compared to 92.8% (CI = 87.3–96) of rural children (Fig. 3). Wildmeat introduction to children’s diets was not associated with household monetary income or maternal education (Supplementary Table S1).

The introduction of other ASFs into children’s diets was similar across rural and urban contexts. In both, most infants had started eating fish (rural = 76.9%; urban = 65.8%) and chicken (rural = 57.7%; urban = 61%), whereas beef consumption was rarer at this age (rural = 34.6%; urban = 41.5%).

### Child hemoglobin

For the most vulnerable rural children (classified by monetary income), eating wildmeat more often was correlated with significantly higher hemoglobin concentration; each additional monthly wildmeat meal was associated with a 0.05 g/dL increase (95% CI = 0.003, 0.103) when controlling for child age, malaria infection (prior 12 months), intestinal parasite infection (prior 3 months), municipality, season, and maternal education. We found the same association for the most vulnerable rural children when classifying vulnerability using the multidimensional poverty index (Supplementary Tables S3, S4, Fig. S4). Wildmeat consumption was associated with a mean increase in hemoglobin concentration of 0.25 g/dL for children with whom it was shared in the previous month, based on their mean consumption frequency (5 meals/month). The maximum potential benefit observed was a 1.0 g/dL increase, for the minority of the most vulnerable rural children who consumed 20 wildmeat meals/month. Wildmeat consumption was not associated with hemoglobin concentration in the least vulnerable rural children, or either subpopulation of urban children (for both monetary income and multidimensional poverty classifications; Supplementary Tables S5, S6). The rural children in our sample had lower hemoglobin concentrations than their urban counterparts (Table 1). The mean hemoglobin concentration of rural children, for both the most/least vulnerable sub-populations, was significantly below (considering 95% CI) the 11 g/dL anemia threshold. For both urban sub-populations, confidence intervals for mean hemoglobin concentration overlapped the anemia threshold (Table 1).

### Anemia amongst most vulnerable rural children

**Figure 4:**
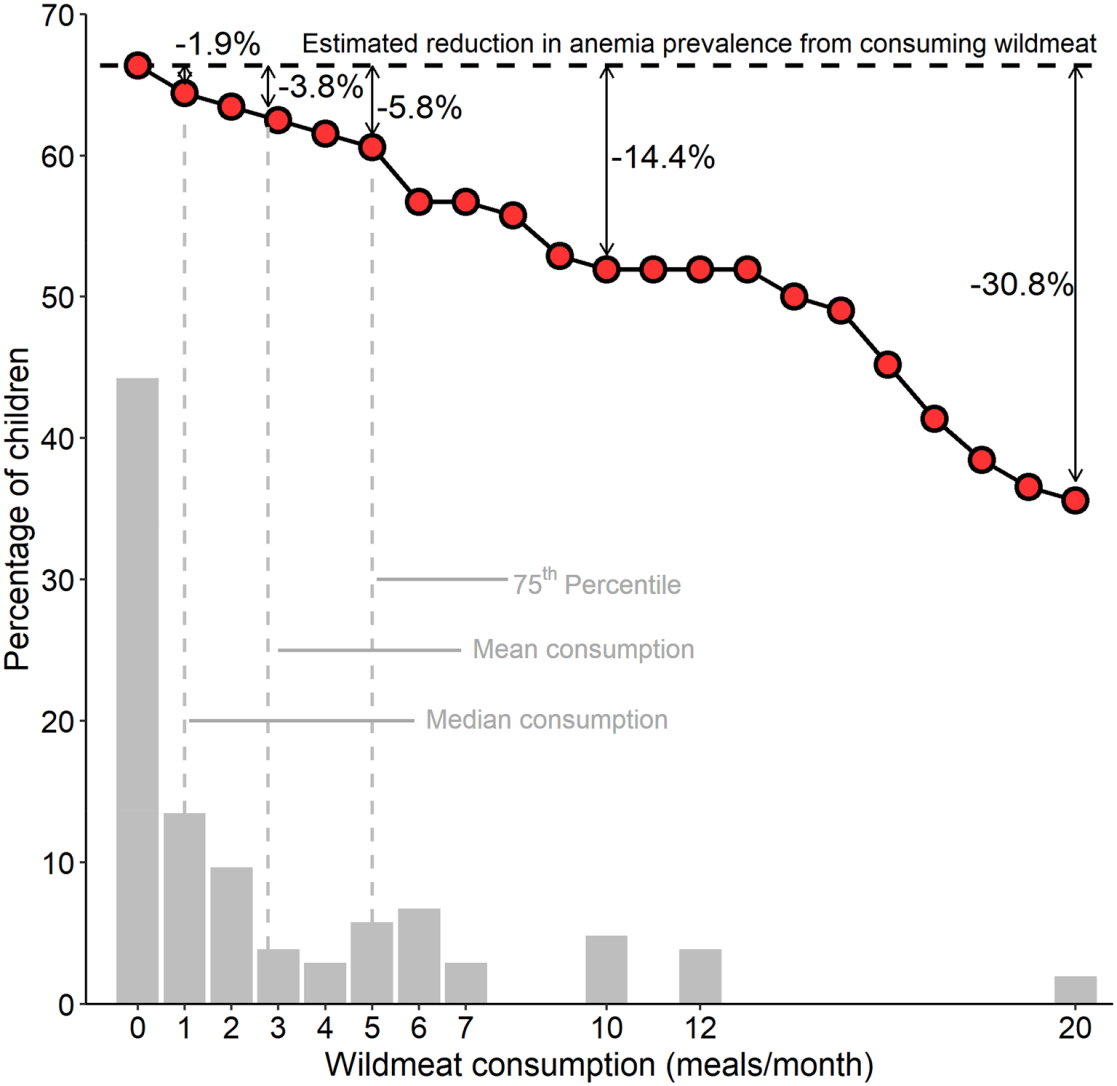
Relationship between frequency of wildmeat consumption and anemia prevalence among vulnerable rural children in Central Amazonia (red dots and black lines). Anemia is defined by hemoglobin concentration < 11 g/dL. These children are classified as vulnerable because their household was one of the poorest (n = 104) 50% of sampled rural households, based on monetary income. Shaded gray bars show the frequency distribution of different levels of wildmeat consumption in this subpopulation. Based on our modeled estimate, each additional meal containing wildmeat increases hemoglobin concentration by 0.05 g/dL (all other control variables kept constant). Twenty wildmeat meals per month was the highest number observed in this subsample. The dotted horizontal line represents the estimated prevalence of anemia if these children were denied access to wildmeat.

In the most vulnerable rural households (based on monetary income) 60.6% of children were anemic. When classified by multidimensional poverty, 68.6% of the most vulnerable rural children were anemic. Those rates were higher than for the least vulnerable rural children (56.9% anemia prevalence) and for both subpopulations of urban children (most vulnerable = 47.1% anemic; least vulnerable = 42.1%) (Table 1). If we assume causality between wildmeat consumption and hemoglobin concentration, denying the most vulnerable rural households (and, hence, their children) access to wildmeat increases anemia prevalence in our empirical sample to 66.3% of these children (95% CI = 61.5%-69.2%), a 9.4% relative increase (i.e., the increase as a percentage of the reference value of 60.6%). The potential increase in anemia is higher when considering multidimensional poverty, with estimated prevalence of 76.2% (95% CI = 73.3%-79.0%), a 11.1% relative increase. Conversely, if all the most vulnerable (based on monetary income) rural children ate wildmeat twice a week (8 meals/month—similar to the current rate of consumption among the least vulnerable rural subpopulation [Table 1]), anemia prevalence in our sample would decrease to 55.7% of these children, equivalent to a 19% relative decrease (Fig. 4). This drop in anemia prevalence from ensuring twice-weekly consumption would be similar when classifying vulnerability based on multidimensional poverty, decreasing to 62.8% of the most vulnerable children compared to the no-wildmeat consumption scenario, equivalent to a 17.6% relative decrease (Supplementary Fig. S4).

Across all 44 highly river-dependent municipalities in Amazonas State, we estimate that in 2019 there were between 43,687 and 64,655 extremely vulnerable (classified by monetary income poverty) rural children aged between 6-months and 5-years-old. These lower and upper estimates equate to between 54 and 80% of young children inhabiting those places. We report this range instead of a single value because we relied on official municipality-specific count data of households within income classes, rather than means or other descriptors (see ‘Methods’ and Supplementary Information). In a policy scenario of denying wildmeat to these children’s households, we calculate that an additional 2,500 to 3,700 vulnerable rural children would become anemic (aggregated across those municipalities), relative to baseline current levels of wildmeat consumption.

## Discussion

Concerns over zoonotic disease transmission and over-harvesting of wildlife populations must be considered alongside tropical forest-dwellers’ rights and the nutritional importance of wildmeat^5,8,46^. Our study addresses whether consuming wildmeat may protect children against anemia, a serious health impairment often caused by iron deficiency. Our findings go beyond research in a single-village in Madagascar^4^, to examine anemia and linkages with wildmeat-sharing practices in multiple urban and rural populations in a highly-forested Amazonian region. We found that wildmeat consumption is associated with higher hemoglobin among the most vulnerable rural children, with the potential to partially protect them from anemia. This finding was robust to classifying vulnerability to poverty based on monetary income or a multidimensional poverty index. If rural Amazonians were denied access to wildmeat, we calculate that the prevalence of early childhood anemia could increase by between 9.4 and 11.1%. This would equate to impaired well-being and risks to lifelong development and health for several thousand already-disadvantaged children living in forested areas, even considering just one Brazilian State (Amazonas). Conversely, if the most vulnerable rural children consumed wildmeat as often as the least vulnerable rural children, anemia prevalence among the former may drop by 19%. The overall nutritional benefits (i.e., looking beyond a single micronutrient) of wildmeat for rural Amazonian children may be greater than the typical benefits to urban children, for three reasons. First, we show that rural households consume wildmeat more often. Second, our results demonstrate that rural caregivers have less access to domesticated ASFs. Third, we find that rural caregivers typically begin sharing wildmeat earlier-on in a child’s life.

### Rural–urban and socioeconomic differences of wildmeat in children’s diets

A major finding was that four-fifths of riverine rural Amazonian children eat wildmeat, at least sometimes, between their first and second birthdays. This compares to only two-in-five similar-aged urban children. In our study area, rural households consume, on average, four times more wildmeat than their urban counterparts^47^, but consume domesticated ASFs less often.

We found that rural children may benefit from feeding practices which typically introduce wildmeat as a complimentary iron-rich food, during infancy. This sharing practice and the greater access to wildmeat is particularly important because rural Amazonian children face myriad health disadvantages and high anemia risks^48–50^. Interestingly, we found no evidence that monetary income or maternal education—both indicative of socioeconomic status—are related to decisions about the timing of wildmeat sharing. This suggests other causes of rural–urban differences, perhaps related to differences in culture (i.e., values and traditions) and habits acquired early in life^19^. This interpretation is supported by our findings. Specifically, rural–urban migrants’ sharing practices were intermediate between rural and urban non-migrant norms (Supplementary Fig. S3). Moreover, the observed positive effect of migrancy on the probability of sharing wildmeat is marginal to (i.e., controlling for) the effects of any migrant/non-migrant differences in income and maternal education (Supplementary Table S1). Although we did not examine underlying social processes, the feeding practices of rural–urban migrants towards their children may reflect rural values and traditions^51^. At the household-scale, wildmeat consumption in this region is strongly influenced by participation in rural-type activities (e.g., agriculture, fishing, forest product extraction), and whether adults identify as rural–urban migrants^47^. Furthermore, remoteness from urban areas partly explains variation in wildmeat consumption among rural households, hinting at the importance of access to domesticated ASFs^47^.

Urban households might be more prone to under-report wildmeat consumption given it is often acquired through the illegal wildmeat trade^47,52^. However, recent methodological research found no evidence that wildmeat consumption is under-reported through direct questioning in small Amazonian towns^53^ comparable in size to three of our study towns. Additionally, other studies in central Amazonia^52,54^, including in medium-sized towns comparable to Maués, report similar rates of urban consumption as we observed. Moreover, urban Amazonians do not seem to perceive wildmeat purchase as a negative behavior^54^.

### Higher hemoglobin concentration linked to wildmeat consumption by the most vulnerable rural children

Although we cannot be certain of a causal effect, our results indicate that, currently, wildmeat may provide a 0.25 g/dL increase in hemoglobin concentration for the most vulnerable rural children who consume it. If children that currently consume wildmeat were denied it, those with hemoglobin levels just above 11 g/dL would have a high risk of becoming anemic. Plausibly, some caregivers may normally share wildmeat with a child but avoid sharing certain species, perhaps due to taboos linked to perceived health-risks of eating particular species^32^. If so, we may have over-estimated children’s consumption of wildmeat meals, and hence the 0.25 g/dL effect would be a conservative estimate. Nonetheless, this potential benefit is below the 0.69 g/dL reported in Madagascar^4^, although their estimate assumes maximum within-sample wildmeat consumption (11 kg/year/person) rather than their observed median value (< 1 kg). Repeating their calculation would quadruple our estimated potential benefits to 1 g/dL, based on 20 wildmeat meals/month. However, this level of consumption was rarely reported in our sample, and it is over four times higher than current consumption of the most vulnerable rural households.

Our published estimates of current *per capita* annual consumption among the rural households we studied^47^ exceed certain estimates of sustainable harvest limits^55,56^. Therefore, the ecological sustainability of harvest required to sustain higher consumption levels requires assessment. Also, the sustainability of hunting varies across locations due to differences in human population densities, hunting practices, and forest cover. Nevertheless, our results indicate that current levels of wildmeat consumption in the most vulnerable rural households appear to influence hemoglobin concentrations among children with whom this food is normally shared. Across central Amazonia, our data show that wildmeat consumption may be protecting thousands of vulnerable rural children from iron-deficiency anemia. This is an important finding given their unreliable access to ASFs other than fish.

We found no evidence that consuming wildmeat significantly alters hemoglobin concentration among the least vulnerable rural children or urban children, albeit many of these children consumed wildmeat. Golden et al.’s research^4^ in rural Madagascar found that higher-income households were less dependent on wildmeat because of better access to alternatives. In our study, chicken was eaten more often in urban areas and by the least vulnerable rural households, compared to the most vulnerable rural households. Yet, unlike typical forested contexts in Madagascar, virtually all rural and urban households in our sample frequently consumed fish. High fish consumption, typically > 50 kg/year/person in rural Amazonia and including dozens of species^35^, provides diverse nutrients and could reduce overall nutritional dependency on terrestrial wildmeat. There is evidence showing a positive association between fish consumption and hemoglobin concentration in Amazonian children, albeit not controlling for confounding factors^38^. In central Amazonia, fish consumption is high across rural and urban populations, though even higher in the former. We only found a link between wildmeat consumption and hemoglobin for the most vulnerable rural children perhaps, in-part, because this subpopulation has relatively poor access to domesticated ASFs. In other words, beyond eating fish, the most vulnerable rural households seem to be relatively dependent on wildmeat for iron intake, despite the least vulnerable rural households consuming wildmeat more often. Although we did not measure the consumption of iron-rich vegetables, they typically contain less bioavailable micronutrients than ASFs^21,22^. Moreover, rural Amazonian diets are based on starchy staples (mainly manioc) and fish, with relatively low consumption of fruits and vegetables^35,36^. Consequently, non-ASFs may therefore provide them with relatively little iron.

Beyond dietary iron, further research is needed into the nutritional importance of wildmeat for tropical forest-dwellers^3^. Wildmeat contains many micro and macronutrients^15^, which may provide broad nutritional benefits for rural Amazonian children, as well as for the sizable minority of vulnerable urban households who rely directly on forest livelihoods, including hunting. Research elsewhere into ASFs shows that beef provides zinc and B12^21^. Yet, for rural forest-dwellers and urban households that seldom eat beef, these nutrients may come mainly from wildmeat. People in wildmeat-consuming households in our sample eat, on average, between 33.5 kg/year/person (rural) and 12.8 kg/year/person of wildmeat (urban)^47^; both above average beef consumption in Amazonas State (10.9 kg/year/person)^57^. Wildmeat’s relative importance might be even greater for our provincial study populations, given the metropolitan bias of these governmental estimates of annual beef consumption. This is because beef consumption is likely to be higher in large, wealthier Amazonian cities such as Manaus. Finally, wildmeat research needs to move beyond assessing species-specific differences in macronutrients^58^ to investigate micronutrient content^59^ and effects of storage, or cooking method.

### Estimated increased in childhood anemia prevalence if denied access to wildmeat

Our results suggest a vital importance of wildmeat for the most vulnerable rural children; a scenario in which they were denied access to wildmeat would increase anemia prevalence by around 10%. That is, without wildmeat in their diets, some of the non-anemic children who currently eat wildmeat would become anemic. In our study region, this equates to wildmeat influencing the health and development of over 3,000 rural children, given IDA is linked to poorer mental, motor, socio-emotional, and neurophysiological functioning^27^. Impaired childhood development perpetuates the rural ‘poverty cycle’ into adulthood, posing further health risks^27^. By certain measures, extremely remote rural locations are the poorest places on Earth^60^. Accordingly, decision-makers should support approaches to maintain and foster the nutritional benefits of wildmeat for vulnerable rural children in Amazonia, and remote areas elsewhere in the forested tropics.

Ensuring equitable access to wildmeat requires recognition of its importance to human health, livelihoods, and food sovereignty of the rural poor^3,7^. However, current environmental regulations in Brazil are contradictory, meaning that many non-indigenous traditional forest communities are denied legal access to wildmeat^8^. Generally, legal access to natural resources is worse for those rural people living outside of sustainable use reserves. Over-hunting threatens wildlife populations in some areas of Amazonas State, and achieving sustainable use is challenging, requiring community organization and cohesion, and support from governmental and other institutions (e.g. for securing land tenure, or technical assistance) ^61^. Encouragingly, there is evidence of sustainable hunting by indigenous peoples and in protected areas^55,56^, and sustained harvest of common species and crop-raiding ‘pests’ (e.g., rodents)^62–64^. Researchers and practitioners argue that sustainably managing terrestrial game is achievable, based on positive examples of community-based management of aquatic and terrestrial resources^66^. Replacing all wildmeat with alternatives such as beef would be ecologically-disastrous for Central Amazonia because it would lead to large-scale conversion of forests into pasture^67^. Consequently, the potential health benefits that wildmeat provides to rural Amazonian children are probably irreplaceable, and supporting vulnerable rural communities to sustainably harvest wildlife is urgent. Although vulnerability and health-risks vary somewhat at the household-scale, wildmeat is a common resource^3^, and collective, community-scale harvest management is therefore essential.

Other nutritional strategies for preventing anemia prevention include iron fortification of staple foods (e.g., wheat, rice) and providing supplements of iron and other micronutrients. However, these strategies face barriers to implementation, such as insufficient political priority, and deficiencies in access to healthcare, education or public health information^68^. The Brazilian government’s National Iron Supplementation Program, running since 2005, has failed to sufficiently reduce anemia prevalence^40^, due to shortcomings including patchy geographic coverage, ineffectual educational activities, and poor adherence^42,69^. Hence anemia remains high in the country^40^, especially among vulnerable populations^42^.

Anemia is multi-factorial and policy-makers should appreciate that, alone, enabling wildmeat consumption will not adequately protect the health of forest-proximate children. Anemia is also caused by parasitic diseases (e.g., malaria, or intestinal parasite infections^48^) and malabsorption of iron in the gastrointestinal tract. In some parts of Amazonia, the bioavailability of dietary iron can also be hampered by the early inclusion of cow’s milk in children’s diets^18^. Overall, preventing iron deficiency, even when anemia is absent, brings positive health impacts, because mild and moderate iron deficiency may impair tissue functioning^28^.

Certain limitations of this study should be stressed. We relied on a cross-sectional design, hence the observed associations do not necessarily represent causal effects. Also, our estimates of how wildmeat consumption affects hemoglobin concentration would be improved by detailed measurement of each child’s wildmeat consumption. For example, by using observational or food diary data, including amounts, species consumed and cooking method. Nonetheless, in the first such study in Amazonia, we have identified an important association between wildmeat and anemia, an indicator of child health. This region is well-recognized for unique role in supporting planetary health, yet the health-risks and vulnerabilities of Amazonian populations are overlooked by policy-makers in Brazil and beyond^70^.

## Conclusions

The importance of wildmeat to the health of tropical forest-dwellers has long-been discussed yet related empirical evidence has been scarce^1,71^. Our study suggests that wildmeat consumption has the potential to partially protect the most vulnerable rural children in Amazonia against anemia, even where fish is widely-available and regularly eaten. This paper’s novel contribution is showing that the nutritional importance of wildmeat varies by rural/urban location, and household-level vulnerability to poverty. We found no evidence of an association between wildmeat consumption and hemoglobin concentration for urban children, or the least vulnerable rural children. These apparent differences across subpopulations of children in our study area may relate to household access to wildmeat and alternative ASFs, and potential sociocultural influences on childhood feeding practices. Potentially, the nutritional benefits to urban children could be enhanced if caregivers were to begin feeding wildmeat to children at a younger age. The current urban tendency to wait until later in childhood may be explained by cultural norms. For rural Amazonians, wildmeat is likely to be a substantial source of energy, protein, and micronutrients by one-year-old. We have shown that the current level of wildmeat typically consumed by vulnerable rural households has the potential to be partially protective against childhood anemia. For many forest-proximate people, especially those in remote locations, domesticated alternatives to wildmeat are neither affordable nor locally available. Instead, rural forest-dwellers tend to either hunt themselves or acquire wildmeat through social networks^63,72^. Hence, wildmeat may indeed provide an irreplaceable ecosystem service^67^, especially given these populations often lack access to even basic public services or programs designed to promote public health and nutrition. Consequently, developing equitable, community initiatives for sustainable wildlife harvesting is crucial for reconciling human health with risks of defaunation, biodiversity loss, and emergence of zoonotic diseases^5,7^. Wildmeat appears to make an important contribution to child health, yet forest-proximate people face myriad disadvantages. Eliminating anemia and other nutrient deficiencies therefore requires major investments to improve public health and reduce vulnerability.

## Materials and methods

### Study area

Our study was carried out in Caapiranga, Maués, Jutaí, and Ipixuna; municipalities in Brazil’s Amazonas State. These municipalities vary in their accessibility to other centers in a hierarchical urban network (e.g., metropoles, state capitals, sub-regional centers)^73^ (Supplementary Fig. S6). The four municipalities also capture heterogeneity in population size (urban populations: approximately 7,000 to 30,000; total populations: 13,000 to 65,000)^74,75^, watershed, travel distance to the state capital Manaus (from 185 to 2,566 km), and access to larger markets and services (private and public). The municipalities also share commonalities: geographical isolation (i.e., from roads), poor public service provision, and low human development (HDI = 0.49 to 0.59). The four towns (each a municipal urban center) are unconnected to other urban centers by road, and rural communities are entirely river-dependent. In each municipality, we followed the official local territorial boundaries of urban and rural areas, defined by municipal law.

### Sampling

In each municipality, we randomly sampled 200 urban households (100: wet-season; 100: dry-season) and 80 rural (40: wet-season; 40: dry-season), totaling 1,111 households (9 fewer Jutaí rural households due to logistical problems). Because our analyses here only include households with young children (6-months to 5 years old), our total sample size varied across municipalities. Of 800 urban households sampled, 291 were included (390 children). Of 311 rural households sampled, 145 were included (220 children). As such, the total sample size was 610 children. Supporting Information contains more details of the sampling design, including random sampling of households.

### Data collection

We collected data on hemoglobin concentration and socioeconomic data from interviews with household heads and each child’s mother or primary caregiver (when the mother lived elsewhere). We interviewed people in the 2015 dry season (August-December) and 2016 wet season (March-July), pre-testing the questionnaire (May–June 2015) in Autazes, another municipality in Amazonas. The survey was coordinated by P.C.T and L.P., conducted together with A.M., J.O., M.A.T.P, M.G.F.S., M.P.F, and another four trained assistants.

### Hemoglobin concentration

We collected children’s hemoglobin concentration data with the portable device HemoCue Hb 201 + Analyzer. Although hemoglobin concentrations are used to define whether a child is anemic (if < 11 g/dL), this method does not identify if anemia is due to iron-deficiency or something else. Due to high rates of iron-deficiency anemia (IDA) compared to other forms of anemia in the region^39,49,76^, and the suitability of the device for fieldwork (especially in remote rural areas), hemoglobin concentration was the measure we obtained for inferring IDA. Similarly, Brazilian national estimates on iron deficiency are mostly based on measurements of hemoglobin concentration^76^, and the American Academy of Pediatrics recommends this measure for diagnosing IDA^77^. Nonetheless, other blood tests are also recommended to screen for iron deficiency (e.g., red blood cells indices and levels of a blood protein, ferritin)^77^.

### Wildmeat consumption frequency

We defined wildmeat consumption frequency as the number of meals containing wildmeat consumed in the household in the previous 30 days. We obtained this number using direct questioning. We then asked the mother/caregiver two questions about each child’s consumption of wildmeat: (1) whether the child had already eaten it; (2) whether the child normally eats wildmeat when available. In the absence of more precise data on child consumption, we assume that children ate wildmeat during all meals consumed in their household if caregivers reported that the child normally eats wildmeat, when available. Some meals may not have been shared with children (e.g., due to beliefs that some species may be less healthy), therefore we may under-estimate the potential health benefits of each wildmeat meal (i.e., due to over-estimating a child’s wildmeat consumption). Although over ten species were consumed in urban and rural areas, urban consumption concentrated on three species–lowland paca (*Cuniculus paca*), tapir (*Tapirus terrestris*), and white-lipped peccary (*Tayassu pecari*), the last two being classified by the IUCN as Vulnerable to extinction. Rural consumption was more evenly distributed, including frequent consumption of howler monkeys (*Alouatta* spp.), brocket deer (*Mazama* spp.), curassow (no id.), agouti (*Dasyprocta* spp.), collared peccary (*Pecari tajacu*) and tortoise (*Chelonoidis* spp.)^47^.

### Data analysis

All analyses were implemented in R 3.5.1^78^. We separated rural and urban children in the analyses due to different potential confounding factors associated with each type of location. We analyzed (1) sharing of wildmeat with children and, (2) the association between children’s hemoglobin concentration and wildmeat consumption. For both types of analyses we used sets of generalized linear mixed-effect models (GLMM), with child as the unit of analysis. We used GLMMs to account for multiple children in the same household, specifying household as a random factor. To account for the nested sampling design in rural areas, we included riverine community as a higher-level random factor in rural models^79^.

For all wildmeat sharing and hemoglobin concentration models, we ran all possible combinations of predictor and control variables (household- and child-level) using the MuMIn package^81^ (Supplementary Table S7). To improve the convergence of the fitting algorithm, we standardized all non-categorical fixed factors so that each had a mean zero and a standard deviation of one^79^. Alternative models in each set were compared through differences in AICc values relative to the first-ranked model (∆AICc)^82^. We considered that a value of ∆AICc ≤ 2 indicates equally plausible models.

### Children’s wildmeat consumption models

We ran logistic models (binomial distribution). The response variable was whether the child ate wildmeat when available in the household (binary variable; yes/no). Only children in households that declared to have consumed wildmeat in the previous 12 months were included (n_rural_ = 183; n_urban_ = 263). Predictor variables were child age, sex, maternal education, monetary income, and, for urban children only, whether either of the household head was a rural–urban migrant. There was no multicollinearity among numeric variables, with low values of variance inflation factor (VIF) for all variables, for both subsets (highest VIF = 1.06) (Supplementary Fig. S8). However, maternal education was significantly lower in rural–urban migrant households (migrants’ mean schooling years = 6.8; non-migrants = 8.4; *P* < 0.001).

### Hemoglobin concentration models

We separated sub-populations of the children most, and least vulnerable to poverty in each type of location (rural and urban). This is because, a priori, we assumed that their vulnerability to anemia differed due to factors related to myriad aspects of poverty/deprivation. We classified a child’s vulnerability to poverty based on their household’s economic characteristics using (a) monetary income poverty thresholds, and (b) an adapted multidimensional Poverty Probability Index (PPI)^45^. While monetary income is the conventional measure for classifying households in poverty and extreme poverty in urban and rural areas (including for enrolling in Brazilian federal social protection programs, such as conditional cash transfers), it ignores the multidimensional nature of poverty. Monetary poverty also does not account for characteristics of traditional rural forest-dwelling societies: (i) reliance on subsistence activities (harvest and cultivation) to meet consumptive needs; (ii) widespread food-sharing (through practices with other households), and limited access to markets, which together reduce the need and opportunities for monetary transactions. Accordingly, we also included PPI, a poverty measure which incorporates a household’s access to services (e.g., years of schooling, formal employment, sanitation) and selected assets (e.g., fridge, motorized vehicle). We refer to the poorest 50% of sampled rural households and their children as the ‘most vulnerable’ and the least-poor 50% of sampled rural households and their children as ‘least vulnerable’. We avoid ‘wealthiest’ given the myriad disadvantages characterizing our study population. The urban sample is also split into the poorest 50% of sampled households (most vulnerable) and the least-poor 50% (least vulnerable). However, absolute levels of monetary and multidimensional poverty differed between rural and urban areas (see Supplementary Information), and therefore these sub-samples are based on relative, not absolute, measures of poverty. In other words, the economic poverty characterizing the most vulnerable rural households is ‘deeper’ (e.g., lower mean income) than for the most vulnerable urban households. We named households ‘vulnerable’ instead of ‘poor’, because poverty measures are not ideal to characterize traditional rural populations in our study context. We acknowledge that our assessment of vulnerability in terms of monetary poverty and access to education, infrastructure, employment, and material assets (measured by the PPI), ignores other sources of vulnerability (e.g., demographic profiles, gender inequalities, violence)^80^.

We ran Gaussian models, with hemoglobin concentration as the continuous response variable. The candidate predictor variable was the number of meals containing wildmeat in the preceding 30 days. For children not yet consuming wildmeat in households that declared consumption, the number of meals was set to zero. We assumed that a child ate wildmeat in all wildmeat meals consumed in their household, if, according to the caregiver, the child normally ate wildmeat when available. We did not consider possible inequitable intra-household allocation (e.g., caregivers withholding wildmeat meals in order to eat more themselves) of food. However, we note there is evidence of, (a) equitable intra-household distribution of food in low- and middle-income countries^83^, (b) we know that mothers in rural Amazonia tend to protect their children from food scarcity^84^. Control variables included were: season (dry/wet), municipality, fluvial travel distance to the nearest urban area (only for rural sample), household members (number), private toilet (yes/no, but omitted for rural models, as only 12 households had one), monthly monetary household income, maternal education (years), domesticated meat and fish consumption (the number of days in which each type was consumed in the preceding seven days), child age (days), malaria incidence (yes/no; prior 12 months), and medical diagnosis of intestinal worms (yes/no during previous 3 months).

Rural caregivers may have under-reported children’s recent infections with malaria or intestinal parasites due to geographic barriers in healthcare access. The potential for under-reporting is likely to be higher in more remote rural areas where healthcare access is most precarious. For instance, in situ diagnosis of malaria infection requires a well-trained microscopist and electricity. If under-reporting of these infections is correlated with higher wildmeat consumption in more remote areas, this could affect the accuracy of the estimated statistical association between wildmeat consumption and hemoglobin concentration. There was no multicollinearity among numeric variables, with low values of variance inflation factor for all variables for all subsets (highest VIF = 1.67) (see Supplementary Information, Supplementary Table S1 and Supplementary Fig. S7 for a more detailed description on variables, their treatment, and collinearity).

### Estimates of anemia avoidance in the study population and study universe

To estimate changes in anemia prevalence in a scenario where the most vulnerable rural children were unable to access wildmeat, we computed the difference between the observed hemoglobin concentration in our sample of this subpopulation (n = 104) and the wildmeat consumption effects estimated by our model (i.e., one wildmeat meal increases hemoglobin concentration by 0.05 g/dL).

To estimate how many rural children in our study universe (44 river-dependent municipalities in Amazonas State unconnected to the road network [Supplementary Fig. S9]) could be affected by any future change in access to wildmeat, first we used governmental census data to estimate the area’s rural population of ‘most vulnerable’ young children (in terms of monetary income, because of data availability) aged between 6-months and 5-years-old. For each municipality we utilized data on the: relative size of the rural and urban populations (percentage of each); total number of children (6-months to 5 years-old); number of households with monthly income comparable to that of our most vulnerable sample. From those values, we calculated the percentage of children and households within our income range of interest, for each municipality’s total population and total number of households. These three variables were obtained from the most recent census (2010 Brazilian Population Census^74^).

Income data from the census are available for classes of *per capita* monetary income per household, taking the Brazilian minimum wage as the reference (e.g. classes include ‘no-income’, ‘up to 1/8 of the minimum wage’, ‘from 1/8 to 1/4 of the minimum wage’, ‘from 1/4 to 1/2 of the minimum wage’). Because we lacked data for comparing the precise income of censused households with our field estimates of income (which we used to classify household vulnerability), we produced two estimates: (i) a lower estimate (of poverty prevalence) only including households with *per capita* monetary income ≤ 1/8 of the minimum wage, (ii) an upper estimate including households with *per capita* monetary income ≤ 1/4 of the minimum wage. We did so because the income threshold for vulnerable to poverty (*per capita* monetary income ≤ 1/5 of the minimum wage) in our rural field data is in-between these limits. To account for population growth since the last census, we adjusted our calculations of each municipality’s population of rural vulnerable children using official population estimates from 2019^75^. The 2019 estimates only include total population size so we applied each municipality’s overall percentage population change (since 2010) to update our 2010 census data on numbers of rural inhabitants, children 6-months to 5 years-old, and households within our target income range (Supplementary Information).

### Ethics

This research was carried out in accordance with rules and guidelines of the Brazilian National Health Council (Resolution 466/12) and the British Sociological Association. It received ethical approval from Brazil’s National Health Research Ethics Committee (CONEP/CNS; protocol 45,383,215.5.0000.0005) and Lancaster University’s Research Ethics Committee (S2014/126). Written free and informed consent was obtained from all interviewees prior to responding to the questionnaire, and from the legal guardian of each child prior to taking a blood sample for measuring hemoglobin (see Supplementary Information).

## Supplementary Information

Supplementary Information.

## References

[1] Milner-Gulland EJ, Bennett EL (2003). Wild meat: The bigger picture. Trends Ecol. Evol..

[2] Van Vliet N (2017). Bushmeat and human health: Assessing the evidence in tropical and sub-tropical forests. Ethnobio. Conserv..

[3] Ingram DJ (2021). Wild meat is still on the menu: Progress in wild meat research, policy, and practice from 2002 to 2020. Annu. Rev. Environ. Resour..

[4] Golden CD, Fernald LCH, Brashares JS, Rasolofoniaina BJR, Kremen C (2011). Benefits of wildlife consumption to child nutrition in a biodiversity hotspot. P. Natl. Acad. Sci..

[5] Roe D (2020). Beyond banning wildlife trade: COVID-19, conservation and development. World Dev..

[6] Zhou W, Orrick K, Lim A, Dove M (2021). Reframing conservation and development perspectives on bushmeat. Environ. Res. Lett..

[7] Cawthorn D-M, Hoffman LC (2015). The bushmeat and food security nexus: A global account of the contributions, conundrums and ethical collisions. Food Res. Int..

[8] Antunes AP (2019). A conspiracy of silence: Subsistence hunting rights in the Brazilian Amazon. Land Use Policy.

[9] Friant S (2020). Eating bushmeat improves food security in a biodiversity and infectious disease “Hotspot”. EcoHealth.

[10] Fa JE, Currie D, Meeuwig J (2003). Bushmeat and food security in the Congo Basin: Linkages between wildlife and people’s future. Environ. Conserv..

[11] Borgerson C, Razafindrapaoly B, Rajaona D, Rasolofoniaina BJR, Golden CD (2019). Food insecurity and the unsustainable hunting of wildlife in a UNESCO world heritage site. Front. Sustain. Food Syst..

[12] Booth H (2021). Investigating the risks of removing wild meat from global food systems. Curr. Biol..

[13] van Vliet N, Nebesse C, Nasi R (2015). Bushmeat consumption among rural and urban children from Province Orientale, Democratic Republic of Congo. Oryx.

[14] Sirén A, Machoa J (2008). Fish, wildlife, and human nutrition in tropical forests: A fat gap?. Interciencia.

[15] Sarti FM (2015). Beyond protein intake: Bushmeat as source of micronutrients in the Amazon. E&S.

[16] Hoffman LC (2012). What is the role and contribution of meat from wildlife in providing high quality protein for consumption?. Anim. Front..

[17] Fa JE (2015). Disentangling the relative effects of bushmeat availability on human nutrition in central Africa. Sci. Rep..

[18] Castro TG, Baraldi LG, Muniz PT, Cardoso MA (2009). Dietary practices and nutritional status of 0–24-month-old children from Brazilian Amazonia. Public Health Nutr..

[19] Mintz SW, Du Bois CM (2002). The anthropology of food and eating. Annu. Rev. Anthropol..

[20] Lokossou YUA, Tambe AB, Azandjèmè C, Mbhenyane X (2021). Socio-cultural beliefs influence feeding practices of mothers and their children in Grand Popo, Benin. J. Health Popul. Nutr..

[21] Murphy SP, Allen LH (2003). Nutritional importance of animal source foods. J. Nutr..

[22] Neumann CG (2003). Animal source foods improve dietary quality, micronutrient status, growth and cognitive function in Kenyan School Children: Background, study design and baseline findings. J. Nutr..

[23] Desalegn A, Mossie A, Gedefaw L (2014). Nutritional iron deficiency anemia: Magnitude and its predictors among school age children, Southwest Ethiopia: A community based cross-sectional study. PLoS ONE.

[24] Safiri S (2021). Burden of anemia and its underlying causes in 204 countries and territories, 1990–2019: Results from the Global Burden of Disease Study 2019. J. Hematol. Oncol..

[25] Vos T (2016). Global, regional, and national incidence, prevalence, and years lived with disability for 310 diseases and injuries, 1990–2015: A systematic analysis for the Global Burden of Disease Study 2015. Lancet.

[26] *Investing in the future: a united call to action on vitamin and mineral deficiencies: global report, 2009*. (Micronutrient Initiative, 2009).

[27] Walker SP (2007). Child development: Risk factors for adverse outcomes in developing countries. Lancet.

[28] Saloojee H, Pettifor JM (2001). Iron deficiency and impaired child development: The relation may be causal, but it may not be a priority for intervention. BMJ.

[29] Neumann C, Harris DM, Rogers LM (2002). Contribution of animal source foods in improving diet quality and function in children in the developing world. Nutr. Res..

[30] Haileselassie M (2020). Why are animal source foods rarely consumed by 6–23 months old children in rural communities of Northern Ethiopia? A qualitative study. PLoS ONE.

[31] Victor R, Baines SK, Agho KE, Dibley MJ (2014). Factors associated with inappropriate complementary feeding practices among children aged 6–23 months in Tanzania: Complementary feeding practices in Tanzania. Matern. Child Nutr..

[32] Morsello C (2015). Cultural attitudes are stronger predictors of bushmeat consumption and preference than economic factors among urban Amazonians from Brazil and Colombia. E&S.

[33] Parry L, Barlow J, Pereira H (2014). Wildlife Harvest and Consumption in Amazonia’s Urbanized Wilderness: Wildlife consumption in urbanized Amazonia. Conserv. Lett..

[34] Chaves WA, Wilkie DS, Monroe MC, Sieving KE (2017). Market access and wild meat consumption in the central Amazon, Brazil. Biol. Conserv..

[35] Dufour DL, Piperata BA, Murrieta RSS, Wilson WM, Williams DD (2016). Amazonian foods and implications for human biology. Ann. Hum. Biol..

[36] Piperata BA (2007). Nutritional status of Ribeirinhos in Brazil and the nutrition transition. Am. J. Phys. Anthropol..

[37] Garcia MT, Granado FS, Cardoso MA (2011). Alimentação complementar e estado nutricional de crianças menores de dois anos atendidas no Programa Saúde da Família em Acrelândia, Acre, Amazônia Ocidental Brasileira. Cad. Saúde Pública.

[38] Marques RC, Bernardi JVE, Dorea CC, Dórea JG (2020). Intestinal parasites, anemia and nutritional status in young children from transitioning Western Amazon. IJERPH.

[39] Granado FS, Augusto RA, Muniz PT, Cardoso MA (2013). Team, the A. S. Anaemia and iron deficiency between 2003 and 2007 in Amazonian children under 2 years of age: Trends and associated factors. Public Health Nutr..

[40] Nogueira-de-Almeida CA (2021). Prevalence of childhood anaemia in Brazil: Still a serious health problem: A systematic review and meta-analysis. Public Health Nutr..

[41] de Souza AA, Mingoti SA, Paes-Sousa R, Heller L (2021). Combination of conditional cash transfer program and environmental health interventions reduces child mortality: An ecological study of Brazilian municipalities. BMC Public Health.

[42] Ferreira HS (2021). Prevalence of anaemia in Brazilian children in different epidemiological scenarios: An updated meta-analysis. Public Health Nutr..

[43] Leite MS (2013). Prevalence of anemia and associated factors among indigenous children in Brazil: Results from the First National Survey of Indigenous People’s Health and Nutrition. Nutr..

[44] WHO, W. H. O. Prevalence of anaemia in children aged 6–59 months (%). https://www.who.int/data/gho/data/indicators/indicator-details/GHO/prevalence-of-anaemia-in-children-under-5-years-(-) (2021).

[45] Schreiner, M. A Poverty Probability Index (PPI®) for Brazil (2008). (2010).

[46] Walzer C (2020). COVID-19 and the curse of piecemeal perspectives. Front. Vet. Sci..

[47] Carignano, T. P., Morsello, C. & Parry, L. Rural-urban mobility influences wildmeat access and consumption in the Brazilian Amazon. *Oryx* (**In press**).

[48] Ferreira MU (2007). Anemia and iron deficiency in school children, adolescents, and adults: A community-based study in Rural Amazonia. Am. J. Public Health.

[49] de Castro TG, Silva-Nunes M, Conde WL, Muniz PT, Cardoso MA (2011). Anemia e deficiência de ferro em pré-escolares da Amazônia Ocidental brasileira: Prevalência e fatores associados. Cad. Saúde Pública.

[50] Cotta RMM (2011). Social and biological determinants of iron deficiency anemia. Cad. Saúde Pública.

[51] Chaves WA, Valle D, Tavares AS, Morcatty TQ, Wilcove DS (2020). Impacts of rural to urban migration, urbanization, and generational change on consumption of wild animals in the Amazon. Conserv. Biol..

[52] El Bizri HR (2020). Urban wild meat consumption and trade in central Amazonia. Conserv. Biol..

[53] Chaves WA, Valle D, Tavares AS, von Mühlen EM, Wilcove DS (2021). Investigating illegal activities that affect biodiversity: The case of wildlife consumption in the Brazilian Amazon. Ecol. Appl..

[54] Chaves WA, Monroe MC, Sieving KE (2019). Wild meat trade and consumption in the Central Amazon, Brazil. Hum. Ecol..

[55] Ohl-Schacherer J (2007). The sustainability of subsistence hunting by matsigenka native communities in Manu National Park, Peru. Conserv. Biol..

[56] Shaffer CA, Yukuma C, Marawanaru E, Suse P (2018). Assessing the sustainability of Waiwai subsistence hunting in Guyana by comparison of static indices and spatially explicit, biodemographic models. Anim. Conserv..

[57] *Pesquisa de orçamentos familiares, 2008–2009*. (IBGE, 2010).

[58] Aguiar JPL (1996). Tabela de composição de alimentos da Amazônia. Acta Amaz.

[59] de Bruyn J (2016). Food composition tables in resource-poor settings: exploring current limitations and opportunities, with a focus on animal-source foods in sub-Saharan Africa. Br. J. Nutr..

[60] World Bank. *Poverty and Shared Prosperity 2020: Reversals of Fortune*. (World Bank, 2020).

[61] Coad, L. M. *et al. Toward a Sustainable, Participatory and Inclusive Wild Meat Sector*. (Center for International Forestry Research (CIFOR) 10.17528/cifor/007046 (2019).

[62] Cowlishaw G, Mendelson S, Rowcliffe JM (2005). Evidence for post-depletion sustainability in a mature bushmeat market. J. Appl. Ecol..

[63] Carignano Torres P, Morsello C, Parry L, Pardini R (2021). Forest cover and social relations are more important than economic factors in driving hunting and bushmeat consumption in post-frontier Amazonia. Biol. Conserv..

[64] Nunes AV, Oliveira-Santos LGR, Santos BA, Peres CA, Fischer E (2020). Socioeconomic drivers of hunting efficiency and use of space by traditional Amazonians. Hum. Ecol..

[65] Freitas CT (2020). Co-management of culturally important species: A tool to promote biodiversity conservation and human well-being. People Nat..

[66] Campos-Silva JV, Peres CA, Antunes AP, Valsecchi J, Pezzuti J (2017). Community-based population recovery of overexploited Amazonian wildlife. PECON.

[67] Nunes AV, Peres CA, de Constantino PAL, Santos BA, Fischer E (2019). Irreplaceable socioeconomic value of wild meat extraction to local food security in rural Amazonia. Biol. Conserv..

[68] Balarajan Y, Ramakrishnan U, Özaltin E, Shankar AH, Subramanian S (2011). Anaemia in low-income and middle-income countries. Lancet.

[69] Mendes MM, e,  (2021). Association between iron deficiency anaemia and complementary feeding in children under 2 years assisted by a Conditional Cash Transfer programme. Public Health Nutr..

[70] Brondízio ES, de Lima ACB, Schramski S, Adams C (2016). Social and health dimensions of climate change in the Amazon. Ann. Hum. Biol..

[71] Ingram DJ (2020). Wild meat in changing times. J. Ethnobiol..

[72] Nunes AV, Guariento RD, Santos BA, Fischer E (2019). Wild meat sharing among non-indigenous people in the southwestern Amazon. Behav. Ecol. Sociobiol..

[73] Parry L (2018). Social vulnerability to climatic shocks is shaped by urban accessibility. Ann. Am. Assoc. Geogr..

[74] IBGE, I. B. de G. e E. Censo Demográfico 2010. (2010).

[75] IBGE, I. B. de G. e E. Estimativas da população residente para os municípios e para as unidades da federação com data de referência em 1o de julho de 2019. (2019).

[76] Cardoso MA, Scopel KKG, Muniz PT, Villamor E, Ferreira MU (2012). Underlying factors associated with anemia in amazonian children: A population-based cross-sectional study. PLOS ONE.

[77] Mattiello V (2020). Diagnosis and management of iron deficiency in children with or without anemia: consensus recommendations of the SPOG Pediatric Hematology Working Group. Eur. J. Pediatr..

[78] R Core Team. *R: The R project for statistical computing.* (2015).

[79] Zuur AF, Ieno EN, Walker N, Saveliev AA, Smith GM (2009). Mixed Effects Models and Extensions in Ecology with R.

[80] Devereux, S. *Social Protection for Rural Poverty Reduction*. Rural Transformations Technical Series 1 (2016).

[81] Barton, K. *Mu-MIn: Multi-model Inference. R Package Version 0.12.2/r18.* (2009).

[82] Burnham KP, Anderson DR, Burnham KP (2002). Model Selection and Multimodel Inference: A Practical Information-Theoretic Approach.

[83] Berti PR (2012). Intrahousehold distribution of food: A review of the literature and discussion of the implications for food fortification programs. Food Nutr. Bull..

[84] Piperata BA, Schmeer KK, Hadley C, Ritchie-Ewing G (2013). Dietary inequalities of mother–child pairs in the rural Amazon: Evidence of maternal-child buffering?. Soc. Sci. Med..

