# Supplementary material for: Wildmeat consumption and child health in Amazonia
Source: Sci Rep. 2022 Apr 6;12:5213. doi: 10.1038/s41598-022-09260-3 (PMC8986765; doi:10.1038/s41598-022-09260-3)
Supplement: Supplementary file 1 — Supplementary Information. [file 41598_2022_9260_MOESM1_ESM.docx]

**Supplementary Information for**

Wildmeat consumption and child health in Amazonia

Patricia Carignano Torres, Carla Morsello, Jesem D. Y. Orellana, Oriana Almeida, André de Moraes, Erick A. Chacón-Montalván, Moisés A. T. Pinto, Maria G. S. Fink, Maíra P. Freire, Luke Parry

*corresponding authors: Patricia Carignano Torres, Luke Parry

Email: [patriciactorres@usp.br](mailto:patriciactorres@usp.br); [luke.parry@lancaster.ac.uk](mailto:luke.parry@lancaster.ac.uk)

**Supplementary Information**

**Materials and Methods.** Additional information on sampling design, data collection, and analyses.

**Sampling design.** In urban areas, sampling was randomized within census sectors (polygons designed to contain a few hundred households, to facilitate administration of the census), in proportion to the number of households in each sector counted during the most recent Brazilian census, in 2010^1^. Hence, our sampling density of households was slightly higher in more densely populated neighborhoods of the four towns we studied and lower in the more sparsely populated neighborhoods. To account for the areal expansion of these towns since the last census, the proliferation of new housing between 2010 and 2015-16 was visually assessed with Google Earth imagery or, when not available (Jutaí only), private satellite imagery. We allocated a proportion of samples to these new housing areas based on the number of houses we visually identified from the images. We selected households in each census sector using ArcGIS 10.3 by first generating points (XY locations) that represented each household along the streets (within a 20-meters radius from the street), masking out uninhabited sections of urban areas (e.g., plazas) to prevent their sampling. The points to be sampled were then randomly selected from the points generated, with the field-team choosing (*in situ*) the household nearest to the sampling point.

In the rural areas, we selected eight riverine communities per municipality in each of the two sampling seasons (i.e., 16 communities per municipality, overall). Communities were located to capture gradients in fluvial travel distance (i.e., remoteness) from urban centers and a diversity of *várzea* floodplain, non-floodplain (*terra firme*) habitat, and communities in / outside of Sustainable Use Reserves. Sampled communities varied from 7 to 249 km from the nearest urban center (Supplementary Fig. S6). We focused only on small communities and, relative to population size, our sample probably over-represents relatively remote communities. Nonetheless, away from the Amazonian main river-stem (named the River Amazonas [downstream of Manaus] and the River Solimões [upstream of Manaus]), rural communities in central Amazonia tend to be small, with only a few larger communities in each municipality. On arrival in each selected rural community, we elaborated a list of all households in the community and randomly selected five households from that list.

**Household income.** Although rural households in Amazonia are usually poor in monetary terms (Supplementary Table S7), there is still a large difference between those in the lower and upper halves of the monetary income distribution. Median household monetary income for the poorest half of rural households in our sample (i.e., bottom 50%), which we named ‘most vulnerable’, was only USD$84.60 per month (based on USD$1.00 = 3.70 Brazilian Reais exchange rate), which is around one-third of the monthly minimum wage in Brazil. The median household income for the upper 50% of our rural sample, which we named ‘least vulnerable’, was somewhat higher (USD$284.05 per month), is slightly above the monthly minimum wage. All of the most vulnerable rural households (poorest half) in our sample would be categorized as living in *extreme* poverty using the World Bank’s definition (*per capita* daily monetary income below USD$1.90, in 2015), while for the least vulnerable (least poor half) in our rural sample, around 70% of households live in extreme poverty based on the World Bank definition.

For urban areas, median household monetary income for the most vulnerable (poorest half) in our sample was around one minimum wage in Brazil (USD$220.30 per month), while for the least vulnerable (wealthiest, or least poor half) in our sample, it was nearly three minimum wages (USD$636.20 per month). In the most vulnerable urban households, the median daily monetary income *per capita* was below the extreme poverty threshold, and residents of 80% of households lived in extreme poverty (World Bank definition). For the least vulnerable (wealthiest or least-poor half) in our urban sample, median daily monetary income *per capita* (USD$3.35) was above the World Bank’s threshold of (non-extreme) poverty of USD$3.20.

**Household poverty probability index (PPI).** This is a multi-dimensional poverty measure that estimates the likelihood that a household is living below the poverty line. We used the index adapted to Brazil^2^. The index is computed from the answers to 10 questions regarding a household’s characteristics and asset ownership, then attributing values to different answers, which are then summed to obtain a PPI value for each household. In this study, we used eight questions because: (i) one of the ten questions does not relate to the local context –‘whether any household member ages 5 to 18 go to private school or pre-school’– because there are no private schools in the study municipalities; (ii) we lacked data on another question –‘how many household members are managers, administrators, professionals in the arts and sciences, mid-level technicians, or clerks’. We also adapted questions about the ownership of different assets to the local context and data available.

The questions we included were: (1) number of members in the household; (2) number of completed years of schooling of the female head; (3) how many household members work as employees with a written contract, or as civil servants for the government or military; (4) number of rooms in the household; (5) how the household disposes of sewage; ownership of: (6) fridge and/or freezer; (7) motorized vehicle; (8) TV satellite dish.

**Data analysis.** We winsorized the values of monetary income at the 98^th^ percentile to limit extreme values and reduce their possible spurious effect^3^.

**Hemoglobin concentrations models.** There was no multicollinearity among numeric variables, with low values of variance inflation factor for all variables for all subsets (highest VIF=1.67). However, consumption of domesticated animal source foods (measured as the sum of beef and chicken meals) was negatively moderately correlated to fish consumption, and monetary income was positively correlated with household size (number of people) (Supplementary Fig. S7).

Malaria incidence was higher in more remote rural communities compared to those nearer the urban center (mean distance was 132.77 km for children that had malaria versus 78.84 km for those who did not; p<0.001), more likely for rural children of less-educated mothers (mean years of maternal schooling was 2.6 for children that had malaria versus 5.1 years for those who did not; p<0.001), and higher among urban children in the most vulnerable households (mean monthly household income was USD$300.80 for children that had malaria versus USD$489.20 for those who did not; p=0.02).

Intestinal parasite infection (IPI) was not correlated with distance to urban center for rural children (mean distance was 86.80 km for children that reported infection and 76.71 km for those who did not; p=0.32), neither to monetary income or mother’s education. We also found no correlation between IPI and those variables for urban children.

Twenty-five children were dropped from this analysis because they lacked a hemoglobin concentration measure. Of these, fifteen children were not at home (single visit in the rural area and after three visits in the urban area), and for nine other children, parents declined to have them tested. One child was excluded due to measurement problems (final n in rural area=206 children; n in urban area=379 children).

**Estimates of anemia avoidance in the study population and study universe.** We aimed to estimate the number of highly vulnerable rural children from 6-months to 5-years-old in our study universe of 44 river-dependent municipalities in Amazonas State unconnected to the road network^4^ (Supplementary Fig. S9). To do so, we use data on monetary income available from the most recent Brazilian census of 2010^1^.

We included all censused rural households with an income range comparable to our sub-sample of the most vulnerable children to estimate the total number of highly vulnerable children in our age range of interest. We included *per capita* earnings for two of the income ranges available from the online census data: (1) between zero and 1/8 of the Brazilian minimum wage *per capita* per month and (2) between zero and 1/4 of the Brazilian minimum wage *per capita* per month. This is because the income threshold for the most vulnerable to poverty (*per capita* monetary income ≤ ⅕ of the minimum wage) in our rural field data is in-between these limits. Estimates were calculated using data on the fraction of the minimum wage *per capita* because that is how data on household monetary income is presented in the publicly census database (e.g., https://www.ibge.gov.br/estatisticas/sociais/populacao/9662-censo-demografico-2010.html?edicao=9672&t=resultados). From the same online database, we calculated the total number of children (from 6-months to 5-years-olds) in the households that felt within those two income ranges. We then calculated the percentage of those highly vulnerable young rural children in from total population (in each of the 44 municipalities). We then used that percentage value to estimate the current population of highly vulnerable rural young children based on the Brazilian government’s national estimate of total population for the year 2019^5^ (i.e., the most recent estimate, by municipality). The 2019 estimate is based on applying state-level population growth rates to the population of each municipality rather than any field sampling by census agents. As the 2019 estimate does not distinguish rural and urban residents, we considered that the rural to urban ratio remained the same as in 2010 (most recent population census) for each municipality. For instance, in 2010, 57.3% of the population of Ipixuna lived in the rural area, which comprised 12,755 rural inhabitants (from a total of 22,254 inhabitants). To estimate the rural population for 2019, we considered that 57.3% of the total estimated population of 29,689 (IBGE’s 2019 estimate) still lived in the rural area, which resulted in 17,016 rural inhabitants.

**Ethics.** We obtained free and informed consent from all interviewees after explaining the research aims and protocols. This occurred prior to administering the questionnaire. Free and informed consent for hemoglobin concentration measures were obtained separately from the mother, father, or another primary caregiver. In rural areas, we first approached the (typically elected) representative of each riverine community and held a community meeting explaining the research aims and protocols before selecting the households for interview.

**SI References**

1. IBGE, I. B. de G. e E. Censo Demográfico 2010. (2010).

2. Schreiner, M. A Poverty Probability Index (PPI®) for Brazil (2008). (2010).

3. Van Kerm, P. V. Extreme incomes and the estimation of poverty and inequality indicators from EU-SILC. *IRISS Work. Pap. Ser.* 57 (2007).

4. Parry, L. *et al.* Social Vulnerability to Climatic Shocks Is Shaped by Urban Accessibility. *Ann. Am. Assoc. Geogr.* **108**, 125–143 (2018).

5. IBGE, I. B. de G. e E. Estimativas da população residente para os municípios e para as unidades da federação com data de referência em 1o de julho de 2020. (2020).


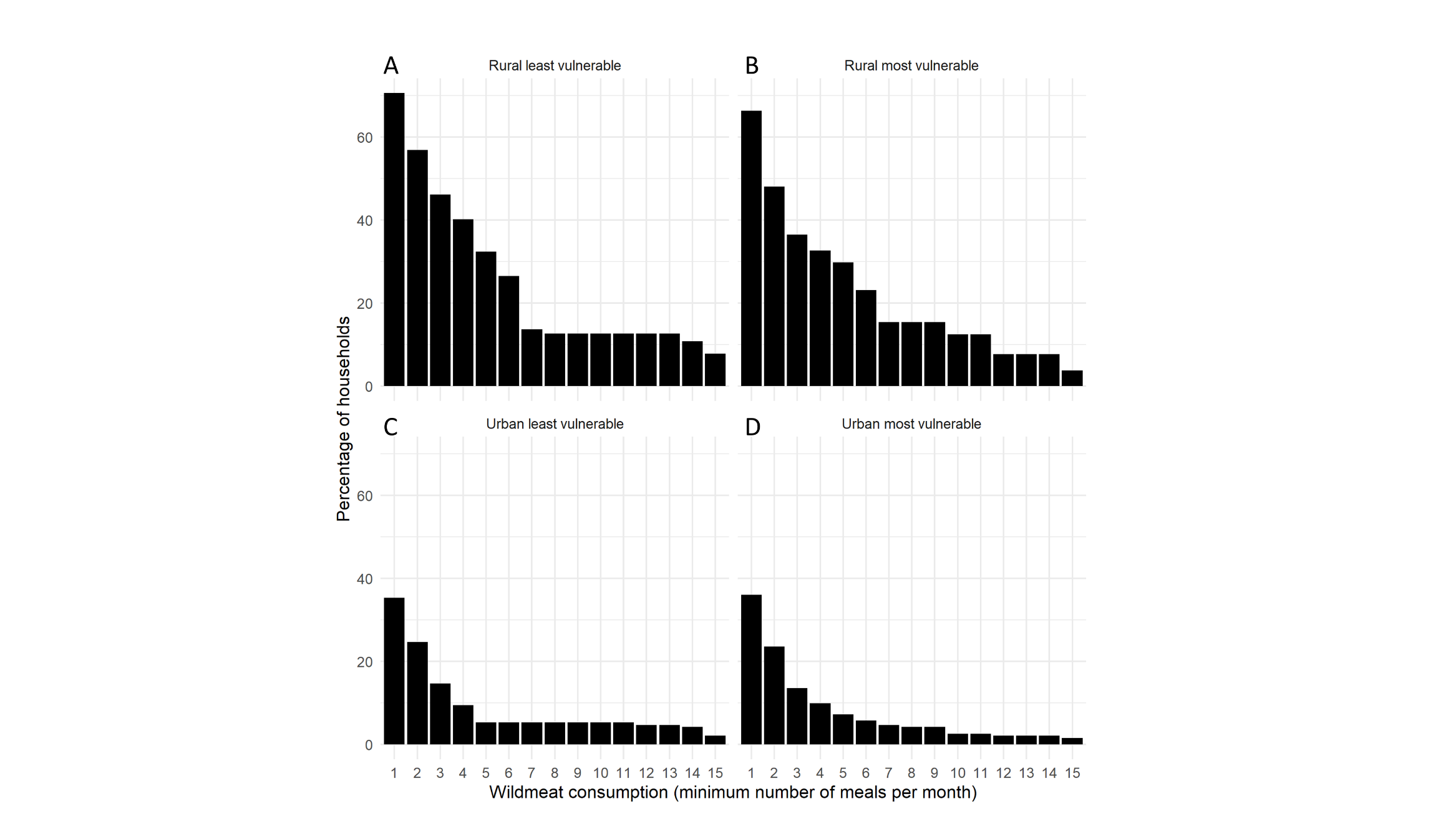


**Supplementary Fig. S1.** Percentage of households we sampled that consumed wildmeat from at least once to at least fifteen times per month over the previous 12 months. (A) Most vulnerable rural households are those with monthly monetary income ≤ USD$128, and (B) least vulnerable rural households >USD$128. (C) Most vulnerable urban households are those with monetary income ≤USD$378, and (D) least vulnerable urban households >USD$378. A conversion rate of USD$1 = 3.70 Brazilian Reais was used.


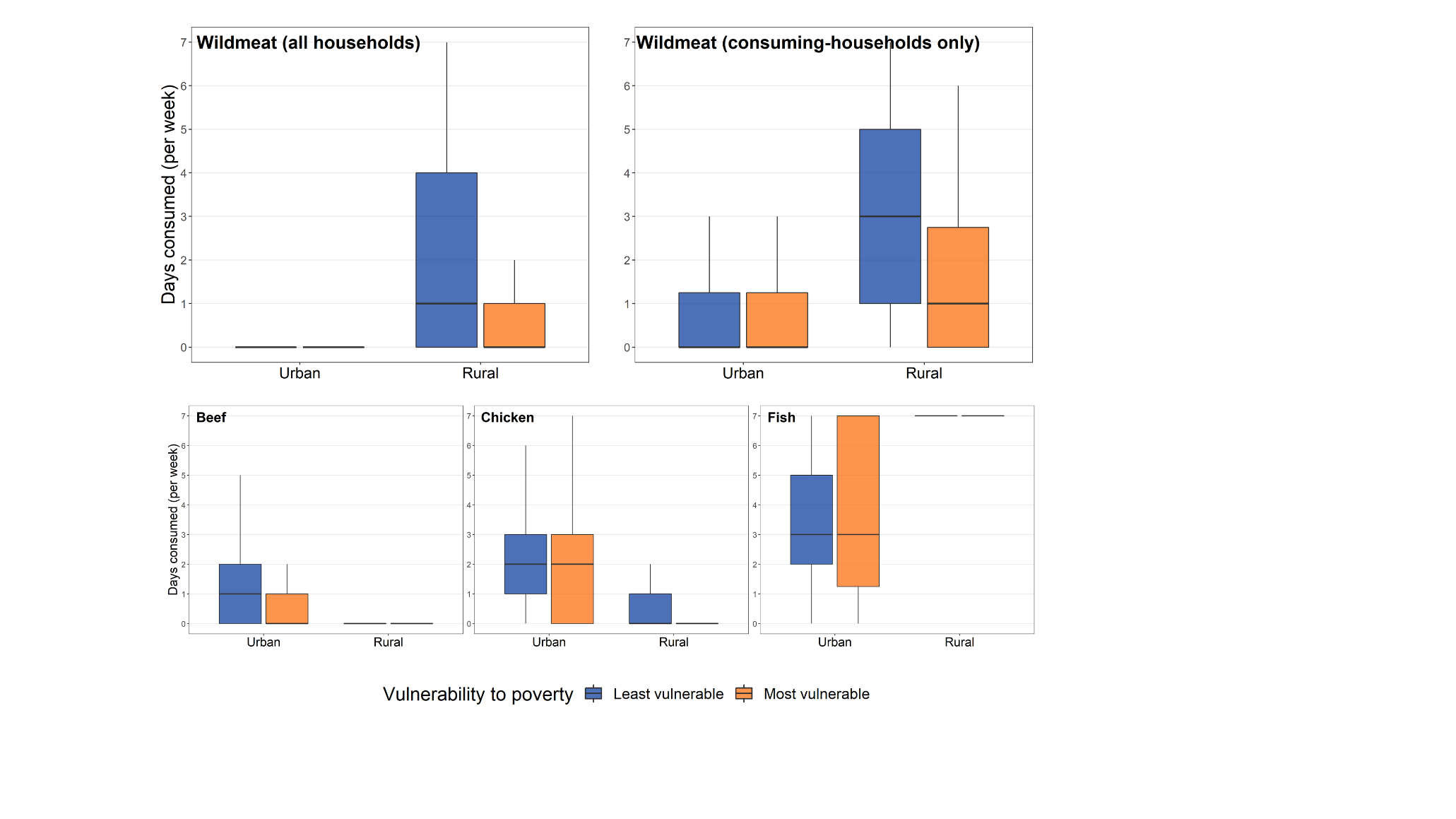


**Supplementary Fig. S2.** Boxplot showing the frequency of consumption (in number of days per week) of different animal source foods in households in rural and urban areas separated by most and least vulnerable sub-populations in the previous seven days. Wildmeat consumption is shown including all households (top left) and only households where wildmeat was consumed in the previous 30 days (top right). Vulnerability to poverty is classified based on thresholds of monetary income. Income levels of the most vulnerable and least vulnerable were ≤USD$128 and >USD$128 (monthly household income) in rural areas and ≤USD$378 and >USD$378 in urban areas. A conversion rate of USD$1 = 3.70 Brazilian Reais was used.


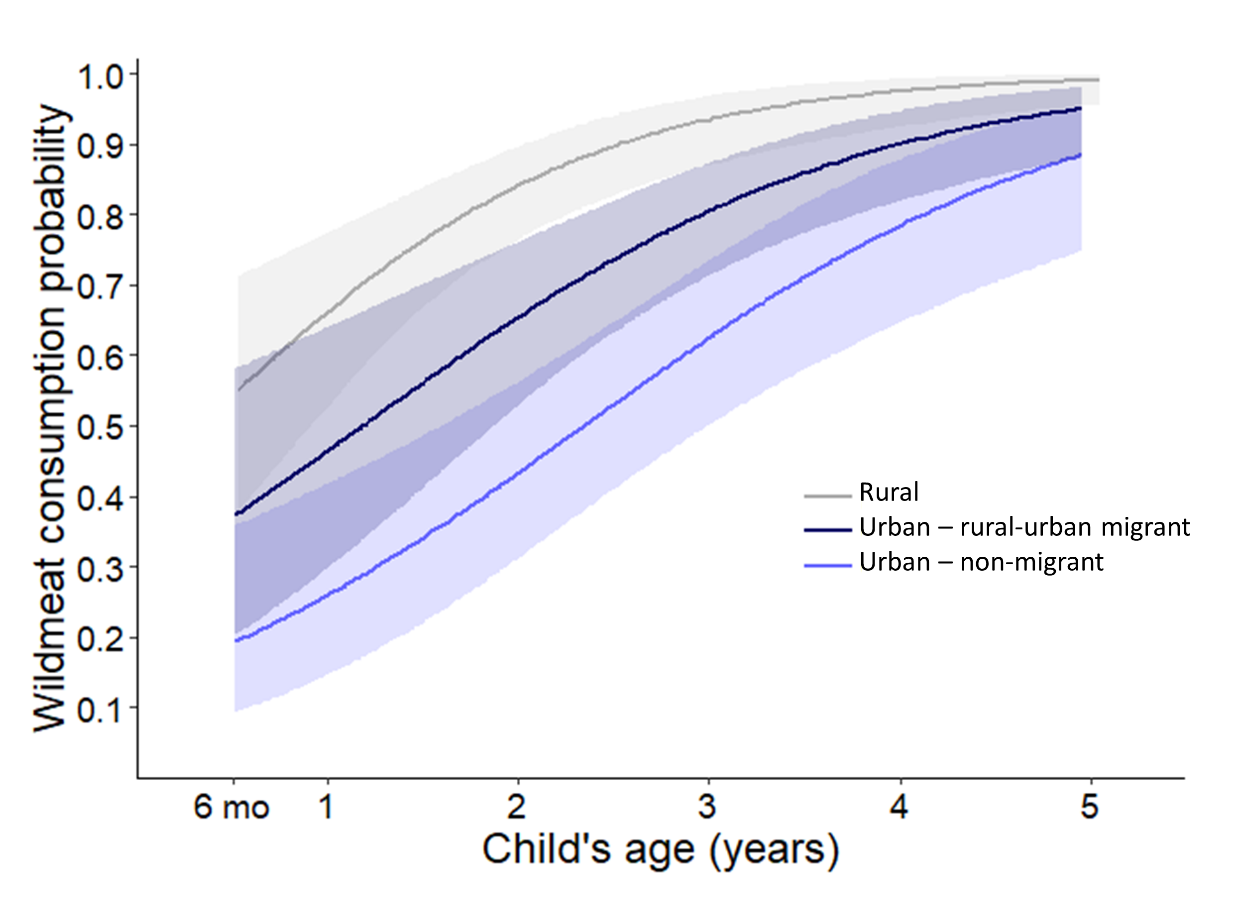


**Supplementary Fig. S3.** Probability of wildmeat consumption by children’s age in rural and urban areas, and in urban households where caregiver(s) identify as a rural-urban migrant(s). Shading shows 95% confidence intervals.


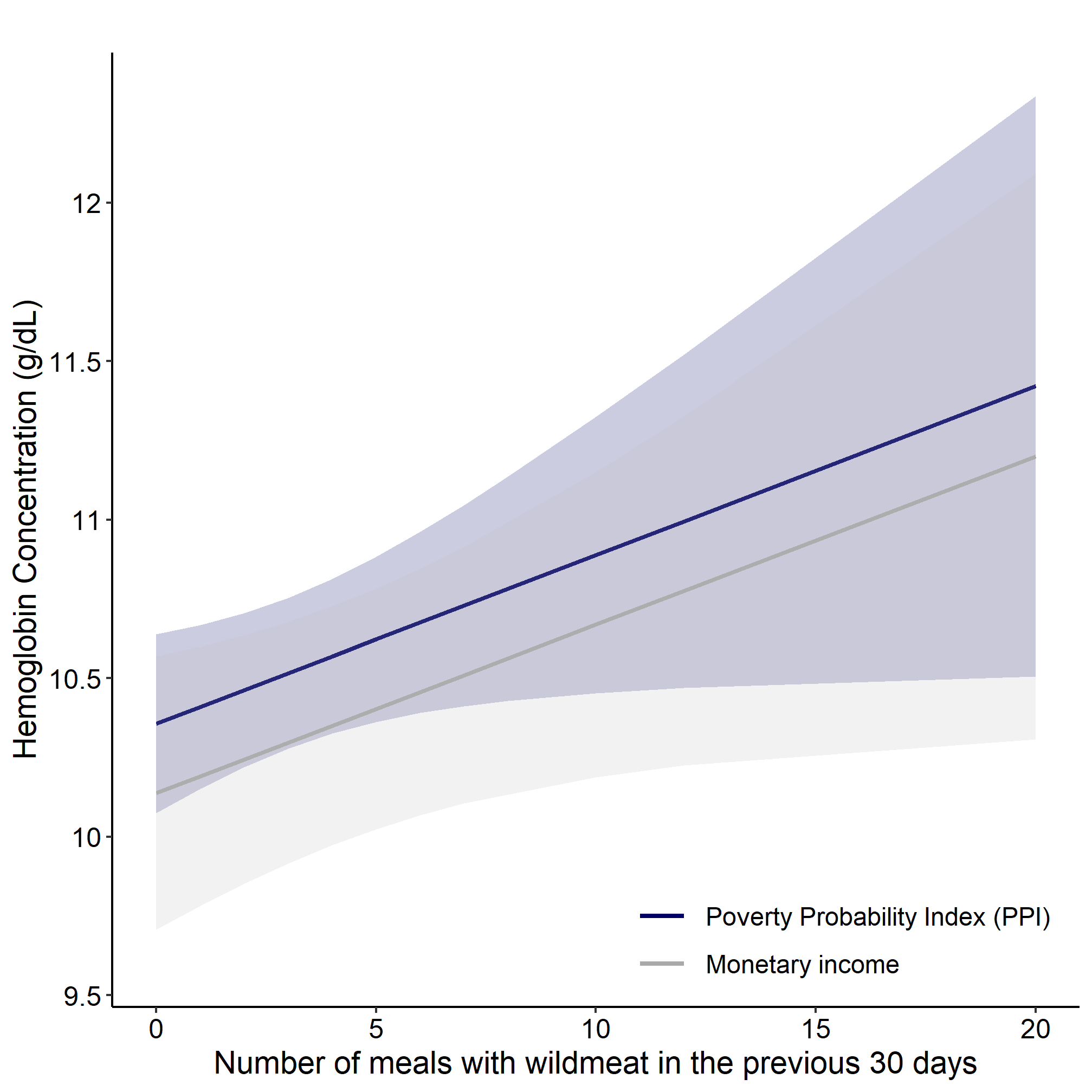


**Supplementary Fig. S4.** Relationship between wildmeat consumption and hemoglobin concentration and for the most vulnerable rural children (classified by monetary income [black line] and the Poverty Probability Index – PPI [gray line]). Shading shows 95% confidence intervals.


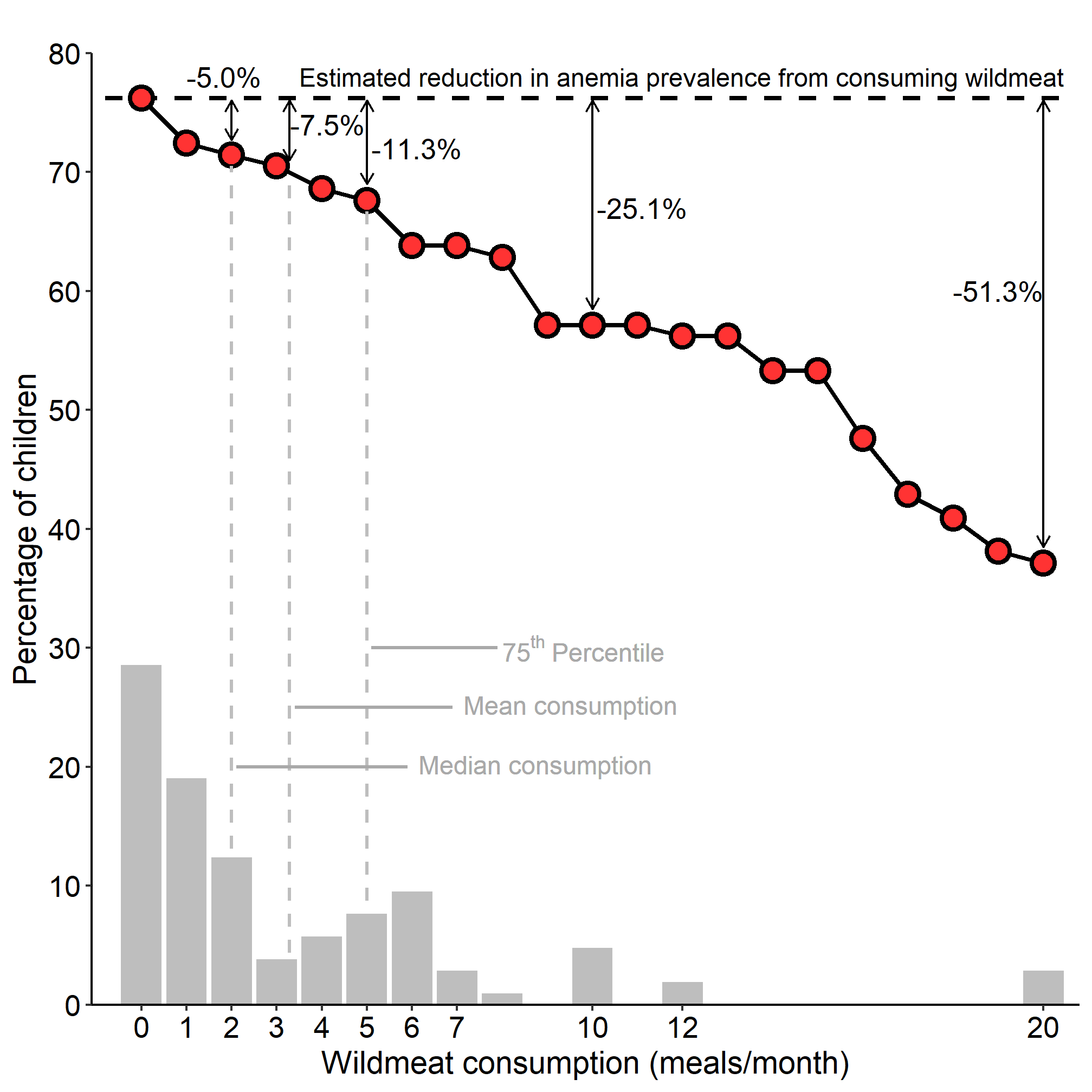


**Supplementary Fig. S5.** Relationship between frequency of wildmeat consumption and anemia prevalence among vulnerable rural children in Central Amazonia (red dots and black lines). Anemia is defined by hemoglobin concentration <11g/dL. These children are classified as vulnerable because their household was one of the poorest (n=104) 50% of sampled rural households, based on multidimensional poverty (PPI). Shaded gray bars show the frequency distribution of different levels of wildmeat consumption in this subpopulation. Based on our modeled estimate, each additional meal containing wildmeat increases hemoglobin concentration by 0.05 g/dL (all other control variables kept constant). Twenty wildmeat meals per month was the highest number observed in this subsample. The dotted horizontal line represents the estimated prevalence of anemia if these children were denied access to wildmeat.


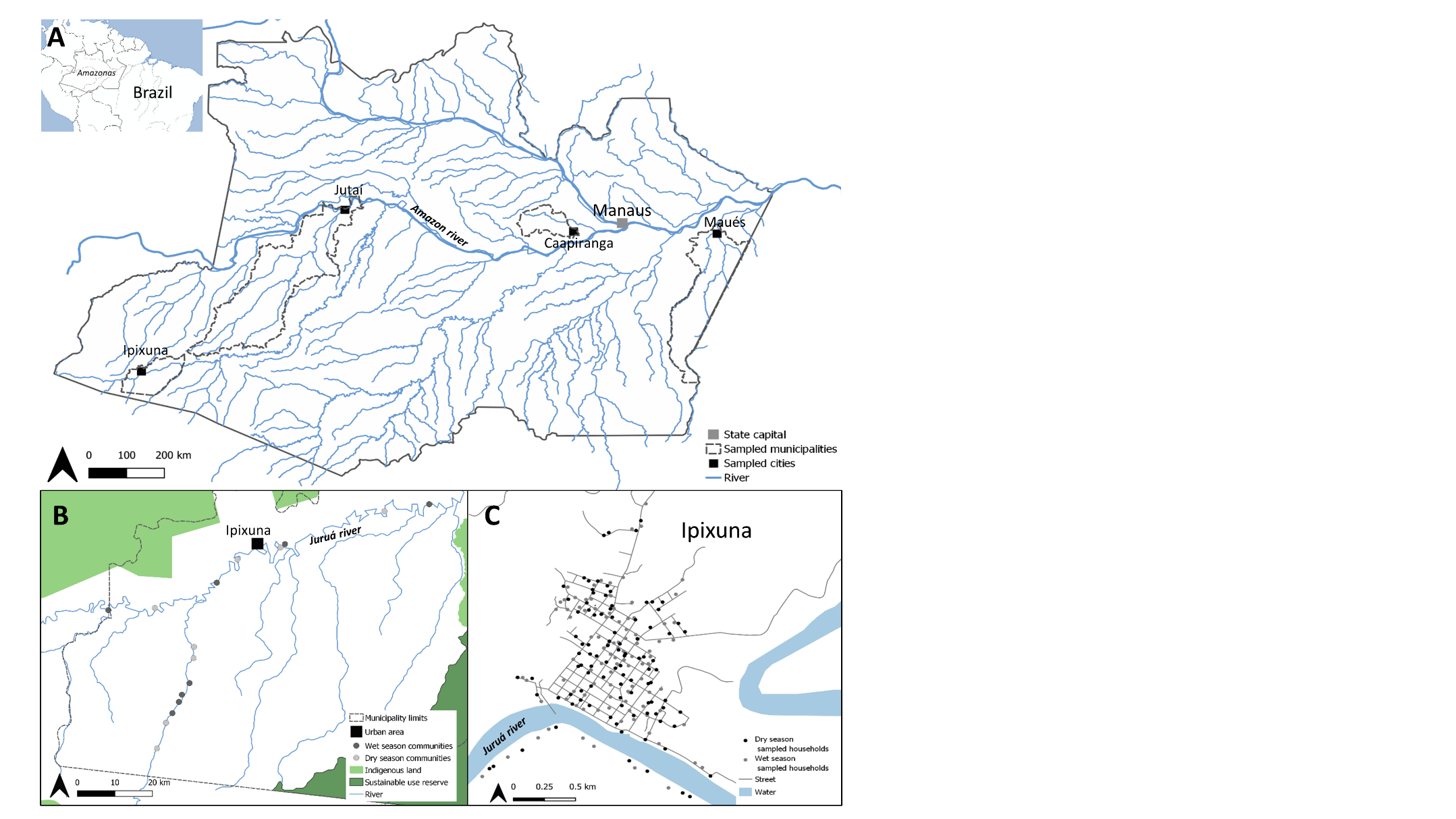


**Supplementary** **Fig. S6.** Study region and sampling design. (A) Location of the study sites in Amazonas state, Brazil; (B) example of the distribution of rural communities sampled in one of the municipalities (Ipixuna); and (C) example of urban households sampled in one of the municipalities (Ipixuna). Map created in QGIS Desktop version 3.16.4 (https://qgis.org/).


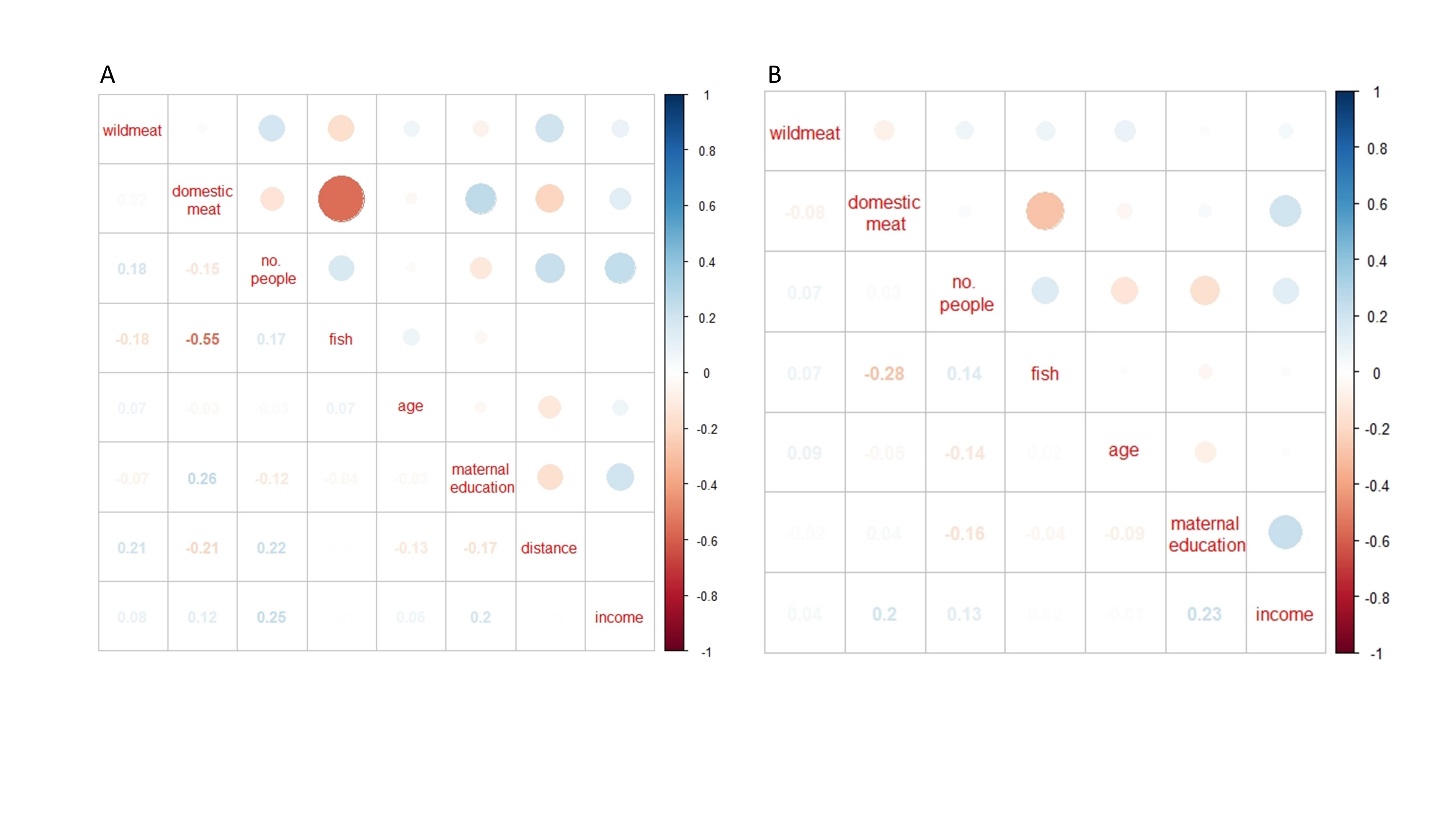


**Supplementary Fig. S7.** Correlations (Pearson) between predictors and control variables for hemoglobin concentration models of rural and urban children. (A) Rural area, (B) urban area. Wildmeat = Number of meals containing wildmeat eaten in the household in the previous 30 days; domestic meat = number of days in which selected animal source foods (beef and chicken) were eaten in the household in the previous seven days; fish = number of days in which fish was eaten in the household in the previous seven days; no. people = number of people living in the household; age = child's age in days; maternal education= number of years of formal schooling taken by the child's mother (or primary caregiver); income =total monetary income earned from salaries, daily work, rent and other forms of remuneration and state transfer (e.g., retirement pension, conditional cash transfers) by all household members in the previous 30 days; distance = fluvial distance in kilometers from the rural community to the municipal urban center.


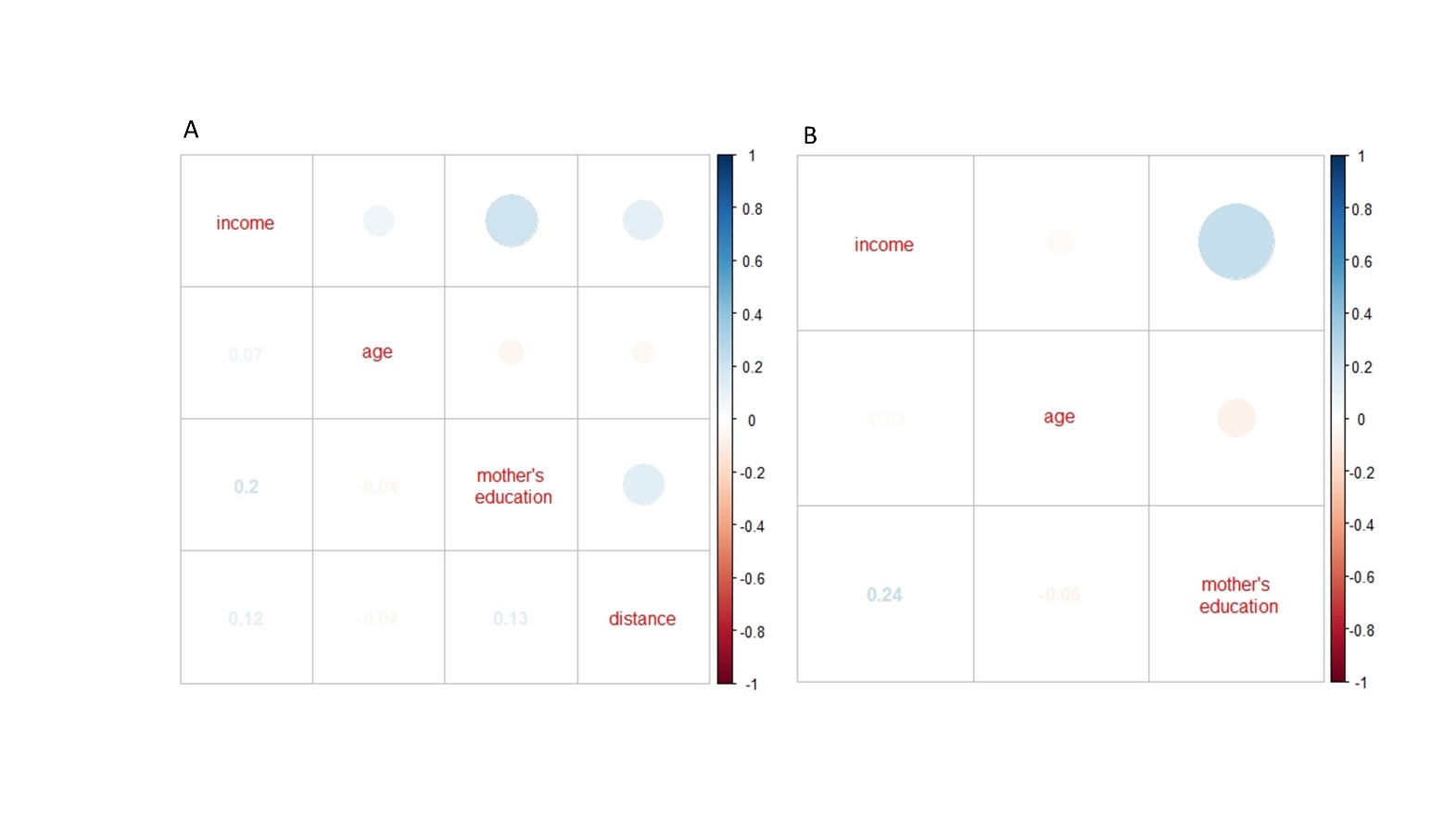


**Supplementary Fig. S8.** Correlations (Pearson) between predictor variables for children’s wildmeat consumption models of rural and urban children. (A) Rural area, (B) urban area. Age = child's age in days; mother’s education = number of years of formal schooling taken by the child's mother (or primary caregiver); income = total monetary income earned from salaries, daily work, rent, and other forms of remuneration and state transfer (e.g., retirement pension, conditional cash transfers) by all household members in the previous 30 days; distance = fluvial distance in kilometers from the rural community to the municipal urban center.


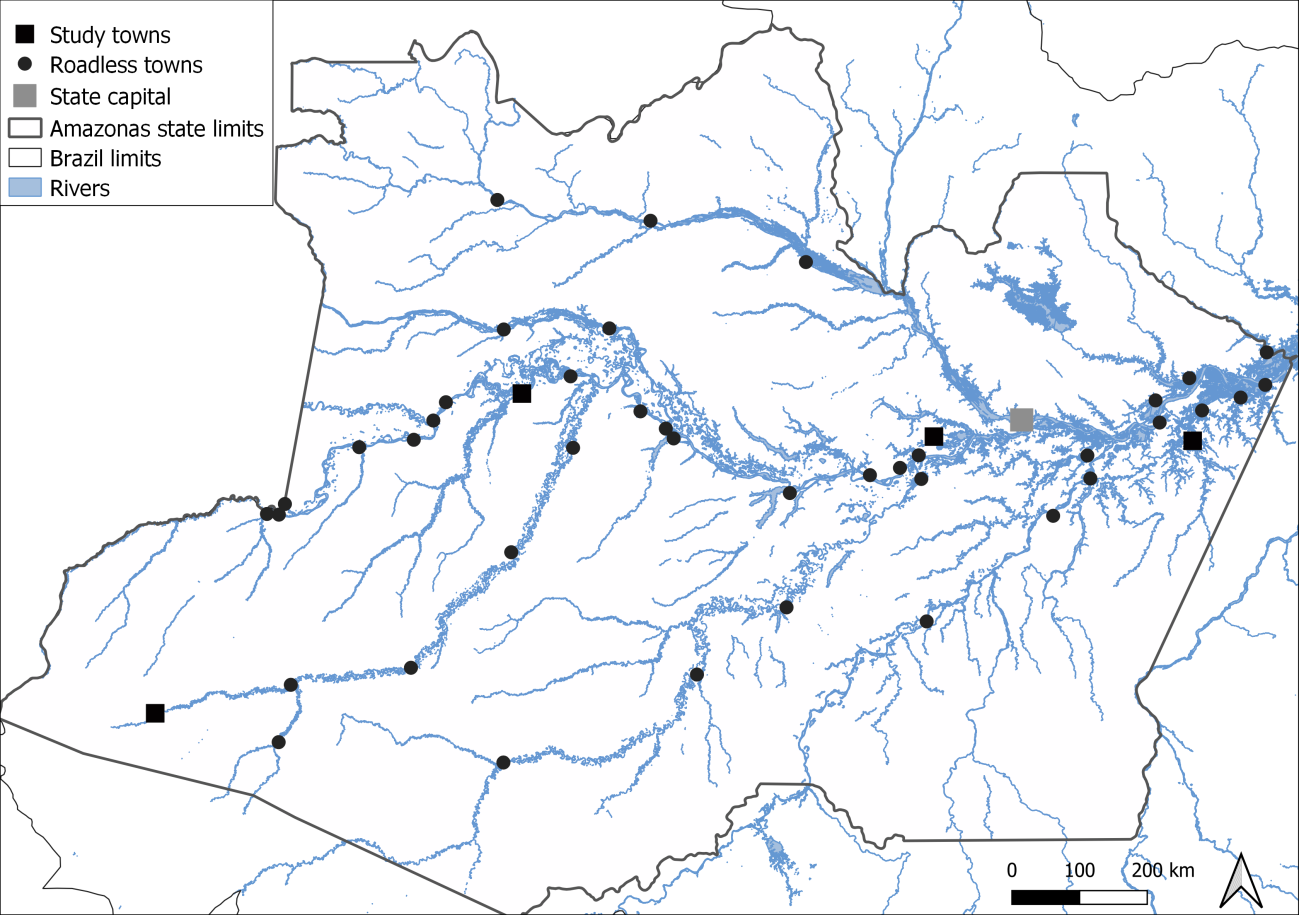


**Supplementary Fig. S9.** Map showing the 44 highly river-dependent towns (unconnected to the road network) in Amazonas State, Brazil. Map created in QGIS Desktop version 3.16.4 ([https://qgis.org](https://qgis.org/)).

**Supplementary Table S1.** GLMM model selection (family=binomial) results for understanding the probability of a child eating wildmeat (i.e., whether caregivers share these meals with a particular child) in rural (n=183 children) and urban (n=263 children) areas. Only selected (equality plausible) models, with ∆AICc < 2, and the intercept-only model for reference, are shown.

| Model | Child’s age  (days) | Sex (male) | Maternal education  (years) | Monetary income  (BRL) | Rural-urban migrant [urban sample only] | K | logLik | AICc | ∆AICc | ωi |
| --- | --- | --- | --- | --- | --- | --- | --- | --- | --- | --- |
| Rural area |  |  |  |  |  |  |  |  |  |  |
| 1 | 1.39 (0.79/1.99) |  |  |  |  | 4 | -71.4 | 151.0 | 0 | 0.20 |
| 2 | 1.40 (0.79/2.01) |  | -0.30 (-0.72/0.13) |  |  | 5 | -70.4 | 151.1 | 0.13 | 0.19 |
| 3 | 1.41 (0.79/2.02) |  |  | -0.10 (-0.56/0.36) |  | 5 | -71.3 | 152.9 | 1.91 | 0.08 |
| Intercept-only |  |  |  |  |  | 3 | -87.0 | 180.1 | 29.15 | 0 |
| Urban area |  |  |  |  |  |  |  |  |  |  |
| 1 | 1.01 (0.57/1.45) |  |  |  | 0.91 (0.23/1.58) | 4 | -169.6 | 347.4 | 0 | 0.29 |
| 2 | 1.03 (0.57/1.48) |  |  | -0.18 (-0.49/0.13) | 0.91 (0.23/1.59) | 5 | -168.9 | 348.1 | 0.76 | 0.20 |
| 3 | 1.01 (0.57/1.45) |  | 0.07 (-0.24/0.39) |  | 0.93 (0.24/1.62) | 5 | -169.5 | 349.3 | 1.91 | 0.11 |
| 4 | 1.01 (0.58/1.45) | -0.10 (-0.69/0.48) |  |  | 0.91 (0.23/1.58) | 5 | -169.5 | 349.3 | 1.95 | 0.11 |
| Intercept-only |  |  |  |  |  | 2 | -194.6 | 393.3 | 45.95 | 0 |

K: number of parameters; logLik: log-Likelihood of the model; AICc: AICc value; ∆AICc: difference in AICc value compared to the first-ranked model; ωi: Akaike weight; coefficients for each variable of the model. Coefficient values are shown for the standardized variables (mean=0; SD=1). 95% CI in parenthesis. The effect of variables was considered significant only if 95% CI did not include zero.

**Supplementary Table S2.** Odds-ratios of the increased likelihood of wildmeat consumption for a one-year increase in child’s age and whether a child lives in a rural-urban migrant household (calculated from GLMM results).

|  | Change in the likelihood of a child consuming wildmeat | | | |
| --- | --- | --- | --- | --- |
| Household type | One year increase in age | | Rural-urban migrant household (yes) | |
|  | Odds-ratio | CI | Odds-ratio | CI |
| Rural children | 2.75 | 1.77 – 4.26 |  |  |
| Urban children in a non-migrant household | 2.19 | 1.56 – 3.08 |  |  |
| Urban children in a rural in-migrant household | 2.47 | 1.26 – 4.85 | 2.48 | 1.26 – 4.85 |

**Supplementary Table S3.** GLMM model selection (family=Gaussian) results for understanding hemoglobin concentration in rural children, with sub-populations of children classified by monetary income (most vulnerable to poverty, n=103 children; least vulnerable, n=102 children). Only selected (equally plausible) models, with ∆AICc < 2, and the intercept-only model for reference, are shown.

| Model | Child’s age (days) | Maternal education (years) | Recent malaria (yes) | Wildmeat meals (per month) | Jutaí | Caapiranga | Maués | Season (wet) | K | logLik | AICc | ∆AICc | ωi |
| --- | --- | --- | --- | --- | --- | --- | --- | --- | --- | --- | --- | --- | --- |
| Most vulnerable |  |  |  |  |  |  |  |  |  |  |  |  |  |
| 1 | 0.35 (0.14/0.55) | 0.31 (0.09/0.53) |  |  | -0.19 (-0.69/0.31) | 0.78 (0.12/1.44) | 0.62 (-0.01/1.26) |  | 9 | -156.4 | 332.8 | 0 | 0.10 |
| 2 | 0.35 (0.14/0.55) | 0.33 (0.11/0.55) |  | 0.22 (0.01/0.42) | -0.11 (-0.61/0.38) | 0.94 (0.28/1.6) | 0.73 (0.09/1.35) |  | 10 | -155.7 | 333.7 | 0.95 | 0.06 |
| Intercept-only |  |  |  |  |  |  |  |  | 4 | -172.1 | 352.7 | 19.90 | 0 |
| Least vulnerable |  |  |  |  |  |  |  |  |  |  |  |  |  |
| 1 | 0.28 (0.08/0.45) |  | -0.99 (-1.73/-0.3) |  |  |  |  |  | 5 | -149.3 | 309.3 | 0 | 0.20 |
| 2 | 0.27 (0.08/0.45) |  | -1.03 (-1.73/-0.3) |  |  |  |  | 0.31 (-0.21/0.85) | 6 | -149.0 | 311.0 | 1.70 | 0.08 |
| Intercept-only |  |  |  |  |  |  |  |  | 3 | -154.7 | 315.7 | 6.41 | 0.01 |

K: number of parameters; logLik: log-Likelihood of the model; AICc: AICc value; ∆AICc: difference in AICc value compared to the first-ranked model; ωi: Akaike weight; coefficients for each variable of the model. Coefficient values are shown for the standardized variables (mean=0; SD=1). 95% CI in parenthesis. The effect of variables was considered significant only if 95% CI did not include zero.

**Supplementary Table S4.** GLMM model selection (family=Gaussian) results for understanding hemoglobin concentration in rural children, with sub-populations classified by the poverty probability index (PPI) (most vulnerable to poverty, n=104 children; least-vulnerable, n=101 children). Only selected (equally plausible) models, with ∆AICc < 2, and the intercept-only model for reference, are shown.

| Model | Child’s age (days) | Maternal education (years) | Recent malaria (yes) | Wildmeat meals (per month) | Household monetary income | K | logLik | AICc | ∆AICc | ωi |
| --- | --- | --- | --- | --- | --- | --- | --- | --- | --- | --- |
| Most vulnerable |  |  |  |  |  |  |  |  |  |  |
| 1 | 0.41 (0.18/0.63) |  | -0.75 (-1.36/-0.15) |  |  | 6 | -160.2 | 333.4 | 0 | 0.07 |
| 2 | 0.41 (0.18/0.63) |  | -0.87 (-1.47/-0.27) | 0.27 (0.01/0.53) |  | 7 | -159.4 | 334 | 0.64 | 0.05 |
| 3 | 0.43 (0.20/0.65) |  | -0.75 (-1.34/-0.15) |  | 0.43 (-0.13/1.01) | 7 | -159.5 | 334.1 | 0.77 | 0.04 |
| 4 | 0.42 (0.20/0.65) |  | -0.86 (-1.46/-0.27) | 0.25 (0.01/0.51) | 0.38 (-0.18/0.94) | 8 | -158.9 | 335.3 | 1.94 | 0.02 |
| 5 | 0.38 (0.16/0.60) | 0.24 (-0.02/0.50) | -0.75 (-1.36/-0.15) | 0.29 (0.04/0.55) |  | 8 | -158.9 | 335.3 | 1.96 | 0.02 |
| Intercept-only |  |  |  |  |  | 4 | -165.4 | 339.2 | 5.86 | 0.00 |
| Least vulnerable |  |  |  |  |  |  |  |  |  |  |
| 1 | 0.34 (0.14/0.55) | 0.32 (0.08/0.55) |  |  |  | 6 | -152.7 | 318.4 | 0 | 0.12 |
| 2 | 0.34 (0.14/0.56) | 0.32 (0.08/0.55) | -0.06 (-1.24/1.08) |  |  | 7 | -152.4 | 320 | 1.61 | 0.06 |
| 3 | 0.36 (0.16/0.58) | 0.33 (0.10/0.57) |  |  | -0.18 (-0.37/0.01) | 7 | -152.4 | 320.1 | 1.7 | 0.05 |
| 4 | 0.29 (0.09/0.50) |  |  |  |  | 5 | -154.8 | 320.2 | 1.83 | 0.05 |
| Intercept-only |  |  |  |  |  | 4 | -157.5 | 323.4 | 5.01 | 0.01 |

K: number of parameters; logLik: log-Likelihood of the model; AICc: AICc value; ∆AICc: difference in AICc value compared to the first-ranked model; ωi: Akaike weight; coefficients for each variable of the model. Coefficient values are shown for the standardized variables (mean=0; SD=1). 95% CI in parenthesis. The effect of variables was considered significant only if 95% CI did not include zero.

**Supplementary Table S5.** GLMM model selection (family=Gaussian) results for understanding hemoglobin concentration in urban children, with sub-populations classified by monetary income (most vulnerable to poverty, n=191 children; least-vulnerable, n=190 children). Only selected (equally plausible) models, with ∆AICc < 2, and the intercept-only model for reference, are shown.

| Model | Child’s age (days) | Recent malaria (yes) | Household size (number of people) | Internal toilet (yes) | Jutaí | Caapiranga | Maués | K | logLik | AICc | ∆AICc | ωi |
| --- | --- | --- | --- | --- | --- | --- | --- | --- | --- | --- | --- | --- |
| Most vulnerable |  |  |  |  |  |  |  |  |  |  |  |  |
| 1 | 0.40 (0.24/0.55) | -0.65 (-1.46/0.17) | -0.25 (-0.44/-0.06) |  |  |  |  | 6 | -296.4 | 605.2 | 0 | 0.14 |
| 2 | 0.38 (0.22/0.53) |  | -0.23 (-0.41/-0.04) |  |  |  |  | 5 | -297.6 | 605.5 | 0.32 | 0.12 |
| 3 | 0.41 (0.26/0.56) |  |  |  |  |  |  | 4 | -299.0 | 606.3 | 1.12 | 0.08 |
| 4 | 0.42 (0.27/0.57) | -0.52 (-1.35/0.30) |  |  |  |  |  | 5 | -298.2 | 606.8 | 1.64 | 0.06 |
| Intercept-only |  |  |  |  |  |  |  | 3 | -308.5 | 625.5 | 20.30 | 0 |
| Least vulnerable |  |  |  |  |  |  |  |  |  |  |  |  |
| 1 | 0.40 (0.23/0.56) |  |  |  | -0.05 (-0.54/0.44) | 0.92 (0.29/1.57) | -0.05 (-0.52/0.42) | 7 | -299.6 | 613.8 | 0 | 0.08 |
| 2 | 0.37 (0.2/0.53) |  | -0.20 (-0.38/-0.02) |  | 0.15 (-0.37/0.66) | 1.04 ( 0.40/1.69) | 0.1 (-0.38/0.57) | 8 | -298.8 | 614.3 | 0.53 | 0.06 |
| 3 | 0.40 (0.2/0.53) | 0.41 (-0.66/1.45) |  |  | 0.18 (-0.34/0.71) | 1.09 (0.44/1.74) | 0.14 (-0.35/0.64) | 8 | -299.1 | 615.0 | 1.17 | 0.05 |
| 4 | 0.41 (0.24/0.57) |  |  | -0.34 (-0.91/0.24) | -0.07 (-0.56/0.42) | 0.96 (0.33/1.61) | -0.03 (-0.49/0.44) | 8 | -299.2 | 615.3 | 1.47 | 0.04 |
| 5 | 0.37 (0.2/0.53) | 0.41 (-0.66/1.45) | -0.20 (-0.38/-0.02) |  | 0.18 (-0.34/0.71) | 1.09 (0.44/1.74) | 0.14 (-0.35/0.64) | 9 | -298.2 | 615.3 | 1.56 | 0.04 |
| 6 | 0.38 (0.21/0.54) |  | -0.20 (-0.38/-0.03) | -0.38 (-0.95/0.18) | 0.13 (-0.38/0.64) | 1.09 (0.45/1.73) | 0.13 (-0.35/0.6) | 9 | -298.2 | 615.4 | 1.64 | 0.04 |
| Intercept-only |  |  |  |  |  |  |  | 3 | -313.3 | 632.7 | 18.90 | 0 |

K: number of parameters; logLik: log-Likelihood of the model; AICc: AICc value; ∆AICc: difference in AICc value compared to the first-ranked model; ωi: Akaike weight; coefficients for each variable of the model. Coefficient values are shown for the standardized variables (mean=0; SD=1). 95% CI in parenthesis. The effect of variables was considered significant only if 95% CI did not include zero.

**Supplementary Table S6.** GLMM model selection (family=Gaussian) results for understanding hemoglobin concentration in urban children, with sub-populations classified by the poverty probability index (PPI) (most vulnerable to poverty, n=181 children; least-vulnerable, n=198 children). Only selected (equally plausible) models, with ∆AICc < 2, and the intercept-only model for reference, are shown.

| Model | Child’s age (days) | Recent malaria (yes) | Internal toilet (yes) | Intestinal parasite infection (yes) | Jutaí | Caapiranga | Maués | K | logLik | AICc | ∆AICc | ωi |
| --- | --- | --- | --- | --- | --- | --- | --- | --- | --- | --- | --- | --- |
| Most vulnerable |  |  |  |  |  |  |  |  |  |  |  |  |
| 1 | 0.52 (0.36/0.68) | -0.21 (-1.45/0.29) |  |  |  |  |  | 4 | -282.4 | 573 | 0 | 0.23 |
| 2 | 0.54 (0.38/0.70) |  |  |  |  |  |  | 5 | -281.5 | 573.3 | 0.29 | 0.20 |
| Intercept-only |  |  |  |  |  |  |  | 3 | -298.7 | 603.6 | 30.6 | 0.00 |
| Least vulnerable |  |  |  |  |  |  |  |  |  |  |  |  |
| 1 | 0.29 (0.13/0.45) |  |  |  | 0.04 (-0.44/0.44) | 0.91 (0.41/1.42) | 0.19 (-0.25/0.64) | 7 | -308.1 | 630.8 | 0 | 0.16 |
| 2 | 0.29 (0.13/0.45) | 0.46 (-0.50/1.41) |  |  | 0.03 (-0.41/0.48) | 0.96 ( 0.44/1.47) | 0.23 (-0.22/0.69) | 8 | -307.5 | 631.7 | 0.9 | 0.10 |
| 3 | 0.30 (0.14/0.45) |  | -0.30 (-0.90/0.31) |  | 0.04 (-0.40/0.49) | 0.97 (0.45/1.48) | 0.25 (-0.20/0.72) | 8 | -307.9 | 632.6 | 1.76 | 0.07 |
| 4 | 0.32 (0.15/0.48) |  |  | -0.26 (-0.63/0.11) | 0.02 (-0.42/0.46) | 0.94(0.43/1.44) | 0.19 (-0.25/0.63) | 8 | -307.9 | 632.6 | 1.77 | 0.07 |
| Intercept-only |  |  |  |  |  |  |  | 3 | -318.4 | 642.9 | 12.1 | 0.00 |

K: number of parameters; logLik: log-Likelihood of the model; AICc: AICc value; ∆AICc: difference in AICc value compared to the first-ranked model; ωi: Akaike weight; coefficients for each variable of the model. Coefficient values are shown for the standardized variables (mean=0; SD=1). 95% CI in parenthesis. The effect of variables was considered significant only if 95% CI did not include zero.

**Supplementary Table S7.** Household- and individual-level characteristics of the study population.

| Variable | Mean (SD or percentage) | | Description |
| --- | --- | --- | --- |
| Household variables | Rural | Urban |  |
| Household size | 7.1 (2.9) | 6.9 (2.9) | Number of people living in the household |
| Total monthly monetary income [median (IQR)]*^,^** | $130.10 ($213.20) | $372.30 ($415.00) | Total monetary income earned from salaries, daily work, rent, and other forms of remuneration and state transfer (e.g., retirement pension, conditional cash transfers) by all household members in the previous 30 days |
| Monthly wildmeat consumption frequency [median (IQR)]* | 2 (6) | 0 (1) | Number of meals containing wildmeat eaten in the household in the previous 30 days |
| Weekly wildmeat consumption frequency (wet season only) [median (IQR)] | 1 (4) | 0 (0) | Number of meals containing wildmeat eaten in the household in the previous seven days |
| Weekly fish consumption frequency [median (IQR)] | 7 (0) | 3 (4) | Number of meals containing fish eaten in the household in the previous seven days |
| Weekly domesticated meat consumption frequency [median (IQR)] | 0 (1) | 3 (4) | Number of meals containing domesticated meat eaten in the household in the previous seven days |
| Maternal education | 4.9 (3.5) | 7.6 (3.7) | Number of years of formal schooling taken by the child's mother (or primary caregiver) |
| Travel distance to the urban center (km) | 85.6 (67.7) | _ | Fluvial distance in kilometers from the rural community to the urban center of the municipality |
| Internal toilet (yes) | 7.7 % | 81 % | Whether the household had at least one toilet facility inside the house |
| Rural-urban migrant (yes) | _ | 64.1 % | Whether one of the household heads migrated from the rural area |
| Individual variables |  |  |  |
| Female | 52 % | 45.9 % | Child's sex |
| Age | 1,008 (502) | 986 (472) | Child's age in days |
| Malaria (yes) | 10% | 3.60% | Whether the child had malaria in the previous 12 months |
| Intestinal worm infection (yes) | 21.8 % | 26 % | Whether the child was diagnosed with intestinal worm infection in the previous three months. |
| Monthly wildmeat consumption frequency [median (IQR)]* | 1 (5) | 0 (0) | Number of meals containing wildmeat eaten by the child in the previous 30 days (equals the number in the household if the child eats wildmeat when available) |
| Hemoglobin concentration (g/dL) | 10.6 (1.2) | 11.1 (1.2) |  |
| Anemia (yes) | 55% | 44.1% | Hemoglobin concentration lower than 11 g/dL |

*IQR – interquartile range. **a conversion rate of $1 = 3.70 Brazilian Reais.
